# Supplementary material for: Transition-metal-free synthesis of pyrimidines from lignin β-O-4 segments via a one-pot multi-component reaction
Source: Nat Commun. 2022 Jun 11;13:3365. doi: 10.1038/s41467-022-30815-5 (PMC9188570; doi:10.1038/s41467-022-30815-5)
Supplement: Supplementary file 1 — Supplementary Information [file 41467_2022_30815_MOESM1_ESM.pdf]

Supplementary Information

Transition-metal-free synthesis of pyrimidines from lignin β-O-4 segments via a one-pot multi-component reaction

Bo Zhang [1,6], Tenglong Guo [1,6], Zhewei Li[2,6], Fritz E. Kühn,[3] Ming Lei[2], Zongbao K. Zhao[4], Jianliang Xiao[5], Jian Zhang[1], Dezhu Xu [1], Tao Zhang [1], Changzhi Li [*1]

[1] CAS Key Laboratory of Science and Technology on Applied Catalysis, Dalian Institute of Chemical Physics, Chinese Academy of Sciences, Dalian 116023, China. Email: licz@dicp.ac.cn

[2] State Key Laboratory of Chemical Resource Engineering, Institute of Computational Chemistry, College of Chemistry, Beijing University of Chemical Technology, Beijing 100029, China.

[3] Molecular Catalysis, Catalysis Research Center and Department of Chemistry, Technical University of Munich, Lichtenbergstr. 4, D-85748, Garching bei München, Germany.

[4] Division of Biotechnology, Dalian Institute of Chemical Physics, Chinese Academy of Sciences, Dalian, 116023, China.

[5] Department of Chemistry, University of Liverpool, Liverpool, L697ZD, UK.

[6] These authors contributed equally to this work: Bo Zhang, Tenglong Guo, Zhewei Li

1. Supplementary Methods 3
1.1 General..3
1.2 Experimental methods..4
Typical procedures of the reactions in Figure 4 4
Typical procedure for the synthesis of meridianin derivative (compound 11) 5
Typical procedure of β-O-4 polymer conversion to 4-(4-(benzyloxy)phenyl)-2,6-diphenylpyrimidine (compound **4p**) 6
Calculation weight yield of product **4a** and **11** according to lignin model compounds amount 7
1.3 Optimization of reaction conditions for pyrimidines synthesis 8
Screening of the reaction parameters...8
The effect of base amount 9
The effect of **3a** amount...10
The effect of the reaction time 11
1.4. Copies of NMR spectra and analytical data 12
1.5. Details of DFT calculations 30
2. Supplementary References 34

1. Supplementary Methods

1.1 General

All lignin model compounds β-hydroxy ether substrates were prepared as described in the literature.[1] The chemicals were obtained commercially and used without further purification. ^{1}H and ^{13}C NMR spectra were recorded on a Bruker DRX-400 spectrometer and all chemical shift values refer to δ_{TMS} = 0.00 ppm or solvent peak was used as internal reference. Catalytic runs were monitored by Agilent 7890 with HP-5 capillary column (30 m × 0.32 mm × 0.25 μm) using mesitylene as an internal standard. Analytical TLC plates, Sigma-Aldrich silica gel 60_{F200} were viewed by UV light (254 nm). Column chromatographic purifications were performed on SDZF silica gel 160. The pyrimidines were purified by column chromatography on silica gel (petroleum ether/ethyl acetate, 9:1) to afford pure products.

1.2 Experimental methods

Typical procedures of the reactions in Figure 4

Typical procedure of Reaction (1) in Figure 4: lignin model compound **1a** (0.4 mmol) NaOH (1.6 mmol), internal standard mesitylene (8 mg), and *t*-AmOH (4 mL) were added in a pressure tube (35 mL). The mixture was sealed and heated to 110 ^{o}C for 1 h. After reaction, the solution was cooled to room temperature, and ethyl acetate (6 mL) was added into the mixture. Then hydrochloric acid (2M) was used to acidify the aqueous solution to pH = 1. The organic phase was analyzed by GC-FID to determine the yield of guaiacol **5a** using mesitylene as an internal standard. Then the solvent was evaporated under reduced pressure, and the crude products were purified by column chromatography using petroleum ether/ethyl acetate (9:1) to obtain 71% yield of acetophenone **6**.

Typical procedure of Reactions (2-4, and 6) in Figure 4: lignin model compound **1a** (0.2 mmol) or acetophenone **6** (0.2 mmol), the primary alcohol **3a** or benzaldehyde **7** (0.2 mmol), benzamidine hydrochloride **2a** (0.1 mmol), NaOH (0.8 mmol), internal standard mesitylene (4 mg), and *t*-AmOH (2 mL) were added in a pressure tube (35 mL). The mixture was sealed and heated to 110 ^{o}C for 20 h under air. After reaction, the solution was cooled to room temperature, and ethyl acetate (3 mL) was added into the mixture. Then hydrochloric acid (2M) was used to acidify the aqueous solution to pH = 1. The organic phase was analyzed by GC-FID to determine the yields of guaiacol **5a** and 2,4,6-triphenylpyrimidine **4a** using mesitylene as an internal standard.

Typical procedure of Reaction (5) in Figure 4: (*E*)-chalcone **8** (0.2 mmol) and benzamidine hydrochloride **2a** (0.1 mmol), NaOH (0.8 mmol), internal standard mesitylene (4 mg), and *t*-AmOH (2 mL) were placed in a pressure tube (35 mL). The mixture was sealed and heated to 110 ^{o}C for 20 h. After reaction, the solution was cooled to room temperature, and ethyl acetate (3 mL) was added into the mixture. Then hydrochloric acid (2M) was used to acidify the aqueous solution to pH = 1. The organic phase was analyzed by GC-FID using mesitylene as an internal standard to afford 59% yield of 2,4,6-triphenylpyrimidine **4a**.

Typical procedure for the synthesis of meridianin derivative (compound 11)

Lignin model compound **1d** (110 mg, 0.4 mmol), guanidine hydrochloride **2g** (19 mg, 0.2 mmol), (1-benzyl-1H-indol-3-yl)methanol **3g** (95 mg, 0.4 mmol), NaOH (64 mg, 1.6 mmol), *t*-AmOH (4 mL) were placed in a pressure tube (35 mL). The mixture was sealed and heated to 110 ^{o}C for 20 h. After reaction, the solution was cooled to room temperature, and ethyl acetate (6 mL) was added into the mixture. The solvent was evaporated under reduced pressure, and the crude products were purified by column chromatography using petroleum ether/ethyl acetate (2:1) to obtain the desired product **10** (56 mg, 69% yield, see Page S27 for ^{1}H and ^{13}C NMR spectra).

A mixture of **10** (41 mg, 0.1 mmol) and *t*-BuOK (80 mg, 0.7 mmol) in 1 mL DMSO was stirred at room temperature under atmospheric oxygen atmosphere for 8 h. Upon completion by TLC monitoring, the reaction was quenched with saturated aqueous NH_4Cl (10 mL), and extracted with EtOAc (3×10 mL). The combined organic phase was dried over anhydrous Na_2SO_4 and concentrated under reduced pressure. Isolation by flash silica gel column chromatography (petroleum ether (30-60 ^{o}C)/diethyl ether = 2:1, v/v) afforded **11** as a white solid (22 mg, 70% yield). The overall yield of **11** based on **2g** is 48%, based on **1d** is 24%. Compound **11** was dissolved in d_6-DMSO and transferred into NMR tube for NMR characterization. NMR spectra of compound **11** can be seen on Page S28. The purity is > 95% according to NMR results.

Typical procedure of β-O-4 polymer conversion to 4-(4-(benzyloxy)phenyl)-2,6-diphenylpyrimidine (compound 4p)

β-O-4 polymer was prepared according to literature.[1,2] 4-Hydroxyacetophenone (**I**) was obtained by depolymerization of β-O-4 polymer over binuclear rhodium complex according to our previous work.[3] In detail, β-O-4 polymer (100 mg), NaOH (32 mg) and the binuclear rhodium catalyst (8 mg, 1 mol%), and H_2O (2 mL) were added into a pressure tube under argon atmosphere. The reaction was performed at 110 °C for 18 h. After cooling to room temperature, hydrochloric acid (1 M) was used to acidify the aqueous solution to PH = 1, which was then extracted with ethyl acetate for three times. Th organic layer was combined, washed with brine and dried over anhydride $MgSO_4$. The solvent was evaporated under reduced pressure. The residue was purified by column chromatography using petroleum ether/ethyl acetate (5:1) to obtain the desired products **I** (75 mg, 75% yield).

Synthesis of compound **II** was based on the previous paper.[4] A mixture of **I** (75 mg, 0.55 mmol), benzylbromide (112 mg, 0.66 mmol), and K_2CO_3 (182 mg, 1.32 mmol) in DMF (5 mL) was stirred at room temperature for 72 h. After reaction, water (5 mL) was added into the solution. After filtration, the filtrate was extracted with diethyl ether and the organic extracts were concentrated and purified by column chromatography using petroleum ether/ethyl acetate (5:1) to obtain the desired products **II** (118 mg, 95% isolated yield).

Compound **II** (118 mg, 0.52 mmol), benzamidine hydrochloride **2a** (41 mg, 0.26 mmol), phenylmethanol **3a** (56 mg, 0.52 mmol), NaOH (84 mg, 2.1 mmol), *t*-AmOH (4 mL) were placed in a pressure tube (35 mL). The mixture was sealed and heated to 110 °C for 20 h. After reaction, the solution was cooled to room temperature, and ethyl acetate (6 mL) was added into the mixture. The solvent was evaporated under reduced pressure, and the crude products were purified by column chromatography using petroleum ether/ethyl acetate (2:1) to obtain the desired product **4p** (99 mg, 92% isolated yield; 66% yield based on β-O-4 polymer, see Page S29 for NMR spectra of **4p**).

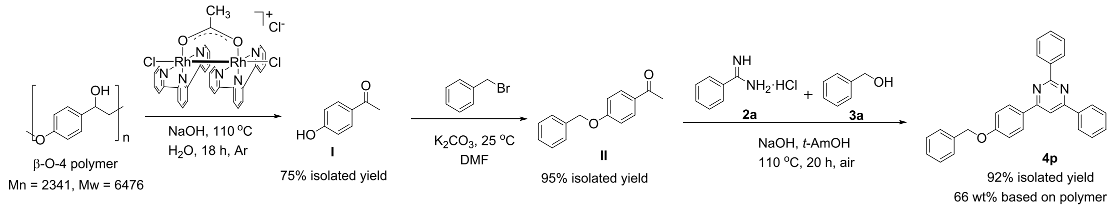

Supplementary Fig. 1. Synthesis of pyrimidine derivative **4p** from conversion β-O-4 polymer.

Calculation weight yield of product 4a and 11 according to lignin model compounds amount

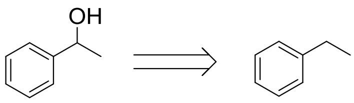

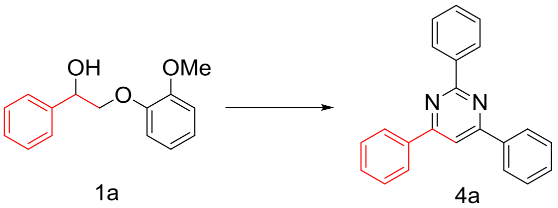

1a
0.4 mmol

4a
0.186 mmol
yield on **2a** is 93%
yield on **1a** is 46.5%.

Part in **1a** that was used for incorporation: $C_8H_{10}O$, M_w 122.

Part that was incorporated: C_8H_{10}, M_w 106. "OH" was "lost".

Not more than 86.9 wt% of lignin model compound could be utilized

(even when the yield and selectivity is 100% and mol ratios are 1:1).

Therefore 0.465 * 0.869 = 40.4 wt% initial lignin model was utilized.

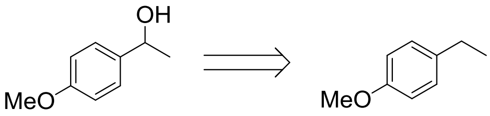

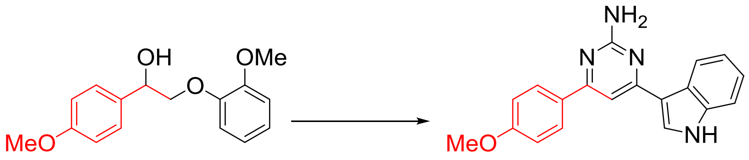

1d
0.4 mmol

11
0.0966 mmol

yield on **2g** is 48%
yield on **1d** is 24%.

Part in **1d** that was used for incorporation: $C_9H_{12}O_2$, M_w 152.

Part that was incorporated: $C_9H_{12}O$, M_w 136. "OH" was "lost".

Not more than 89.5 wt% of lignin model compound could be utilized (even when the yield and selectivity is 100% and mol ratios are 1:1) Therefore 0.24 * 0.895 = 21.5 wt% initial lignin model was utilized.

Supplementary Fig. 2. Calculation weight yield of products **4a** and **11**.

1.3 Optimization of reaction conditions for pyrimidines synthesis

Screening of the reaction parameters

Supplementary Table 1. Base-mediated synthesis of pyrimidine **4a** under different conditions. [a]

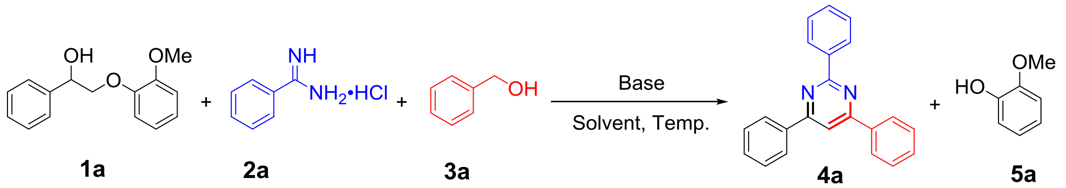

Entry	Base	Solvent	Temp (°C)	**1a** Conv. [%][b]	Yield **4a** [%][b]	Yield **5a** [%][b]
1	--	*t*-AmOH	110	0	0	0
2	NaOH	*t*-AmOH	110	100	95 (93)[c]	99 (95)[c]
3	KOH	*t*-AmOH	110	85	50	82
4	*t*-BuOK	*t*-AmOH	110	100	58	93
5	Cs_2CO_3	*t*-AmOH	110	14	6	3
6	CH_3CH_2ONa	*t*-AmOH	110	2	1	0
7	CaO	*t*-AmOH	110	0	0	0
8	MgO	*t*-AmOH	110	0	0	0
9	$Mg_6Al_2(CO_3)(OH)_{16}$	*t*-AmOH	110	0	0	0
10	NaOH	Dioxane	110	57	36	52
11	NaOH	CH_3CN	110	40	1	24
12	NaOH	DMF	110	7	3	1
13	NaOH	Toluene	110	100	81	93
14	NaOH	*t*-AmOH	90	87	70	83
15	NaOH	*t*-AmOH	70	62	35	58
16[d]	NaOH	*t*-AmOH	110	100	10	14

[a] Conditions: **1a** (0.2 mmol), **2a** (0.1 mmol), **3a** (0.2 mmol), base (8 equiv.), solvent (2.0 mL) in air, t = 20 h; the yields of **4a** and **5a** were calculated based on the amounts of **2a** and **1a**, respectively; [b]The conversion and the yield were determined by GC-FID using mesitylene as an internal standard; [c] Isolated molar yield; [d] Condition: **1a** (0.8 mmol), **2a** (0.4 mmol), **3a** (0.8 mmol), NaOH (3.2 mmol), *t*-AmOH (2.0 mL) in air, t = 20 h.

The effect of base amount

Supplementary Table 2. Screening of base amount for pyrimidines synthesis from lignin β-O-4 model compound.[a]

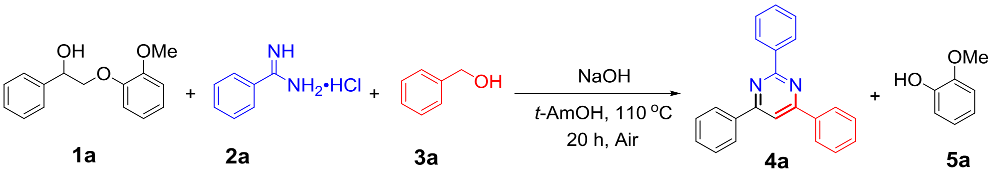

Entry	Base (x equiv.)	**1a** Conv.[b]	Yield **4a**[b]	Yield **5a**[b]
1	8	100	95	99
2	6	83	60	71
3	4	49	25	32
4	2	9	4	8
5	0	--	--	--

[a] Conditions: **1a** (0.2 mmol), **2a** (0.1 mmol), **3a** (0.2 mmol), base (x equiv.), *t*-AmOH (2.0 mL) in air, t = 20 h; the yields of **4a** and **5a** were calculated based on the amounts of **2a** and **1a**, respectively; [b]The conversion and the yields were determined by GC-FID analysis using mesitylene as an internal standard.

The effect of 3a amount

Supplementary Table 3. Screening of **3a** amount for pyrimidines synthesis from lignin β-O-4 model compound.[a]

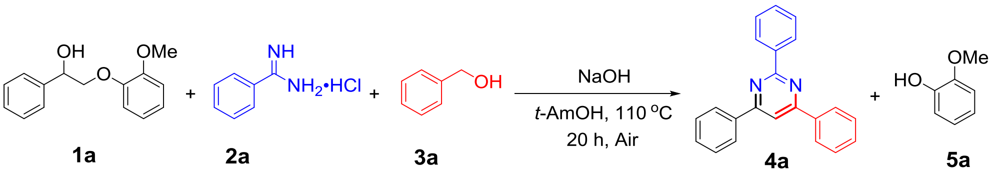

Entry	**3a : 2a**	**1a** Conv. [b]	Yield **4a**[b]	Yield **5a**[b]
1	1:1	86	50	24
2	1.5:1	100	75	72
3	2:1	100	95	99

[a] Conditions: **1a** (0.2 mmol), **2a** (0.1 mmol), **3a** (x mmol), NaOH (0.8 mmol), *t*-AmOH (2.0 mL) in air, t = 20 h; the yields of **4a** and **5a** were calculated based on the amounts of **2a** and **1a**, respectively; [b]The conversion and the yields were determined by GC-FID analysis using mesitylene as an internal standard.

The effect of the reaction time

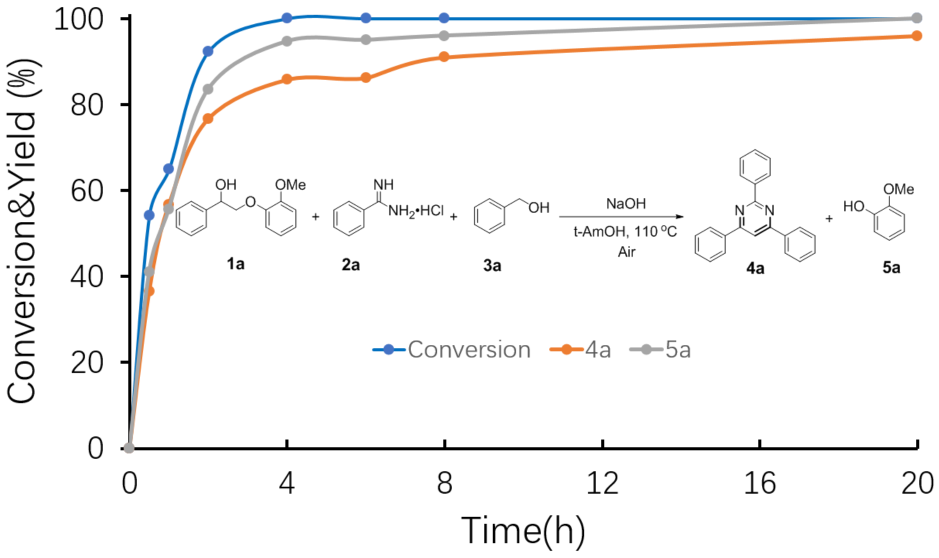

Supplementary Fig. 3. The time course profile of pyrimidine synthesis

1.4. Copies of NMR spectra and analytical data

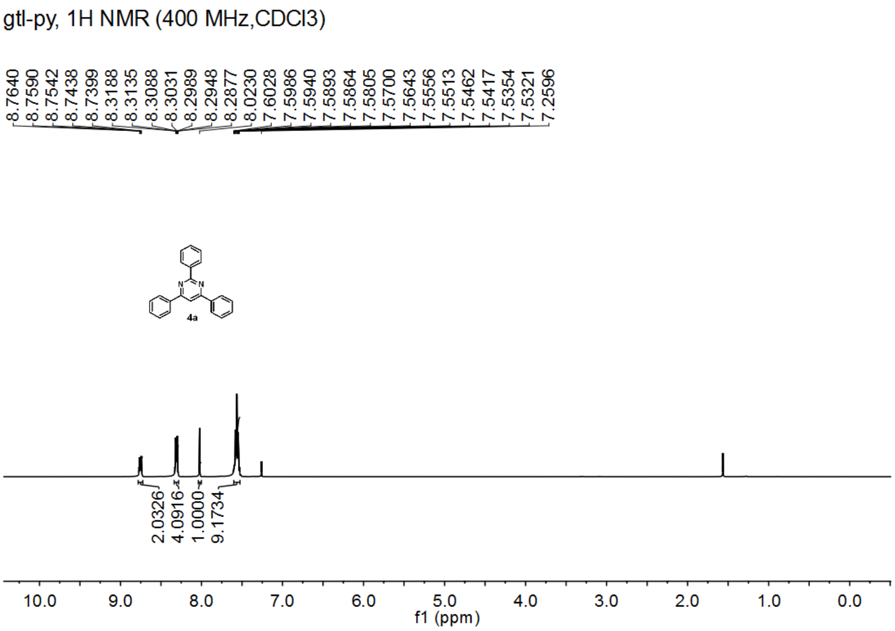

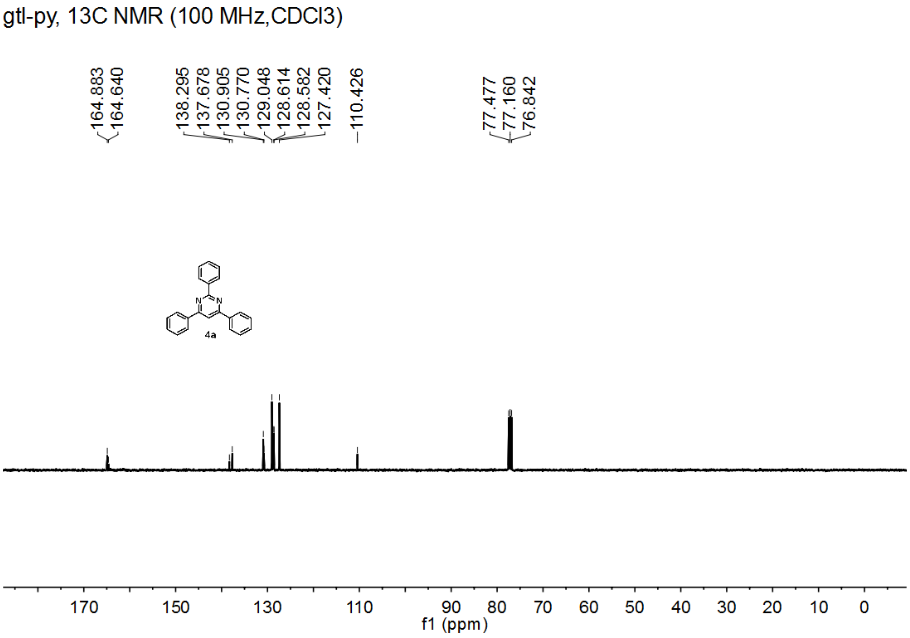

2,4,6-Triphenylpyrimidine (4a): White solid. ^{1}H NMR (400 MHz, $CDCl_3$) δ 8.74 (m, 2 H, aromatic CH), 8.31 (m, 4 H, aromatic CH), 8.02 (s, 1 H, aromatic CH), 7.57 (m, 9 H, aromatic CH). ^{13}C NMR NMR (100 MHz, $CDCl_3$) δ 164.9, 164.6, 138.3 and 137.7 (Cq each), 130.9, 130.8, 129.1, 128.6, 128.6, 127.4, 110.4 (CH).

Supplementary Fig. 4. ^{1}H NMR and ^{13}C NMR spectra for **4a** (Fig. 2 Entry 1).

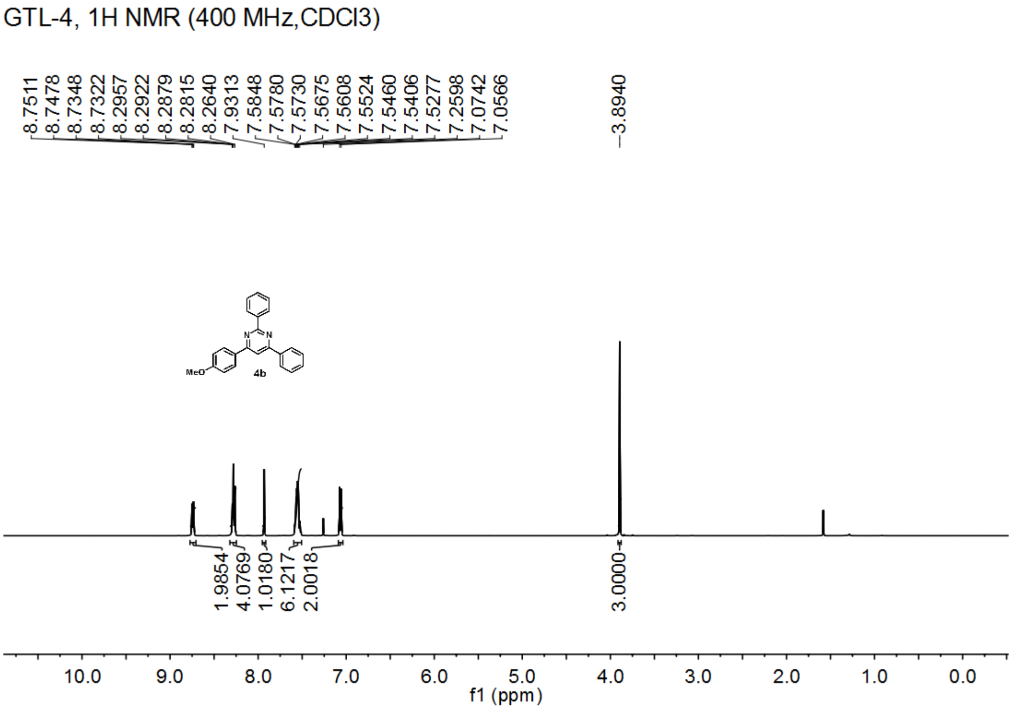

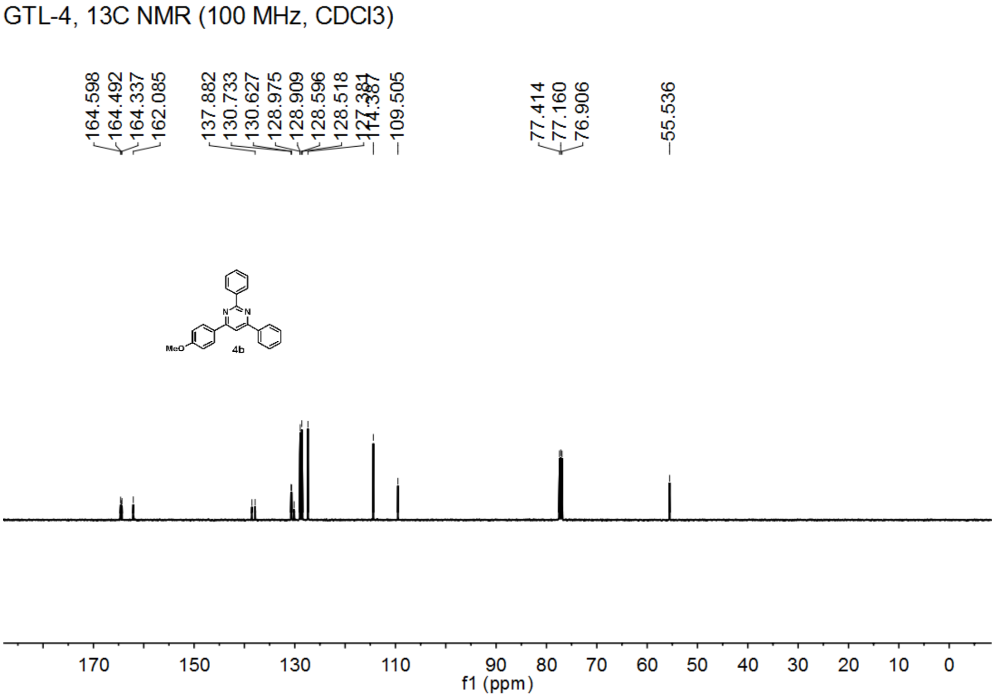

4-(4-Methoxyphenyl)-2,6-diphenylpyrimidine (4b): White solid. ^{1}H NMR (400 MHz, $CDCl_3$) δ 8.74 (m, 2 H, aromatic CH), 8.28 (m, 4 H, aromatic CH), 7.93 (s, 1 H, aromatic CH), 7.60-7.51 (m, 6 H, aromatic CH), 7.07 (d, J = 8.8 Hz, 2 H, aromatic CH), 3.89 (s, 3 H, OCH_3). ^{13}C NMR (100 MHz, $CDCl_3$) δ 164.6, 164.5, 164.3, 162.1, 138.5, 137.9 and 130.1 (Cq each), 130.7, 130.6, 129.0, 128.9, 128.6, 128.5, 127.4, 114.4 and 109.5 (CH), 55.5 (O*C*H_3).

Supplementary Fig. 5. ^{1}H NMR and ^{13}C NMR spectra for **4b** (Fig. 2 Entry 4).

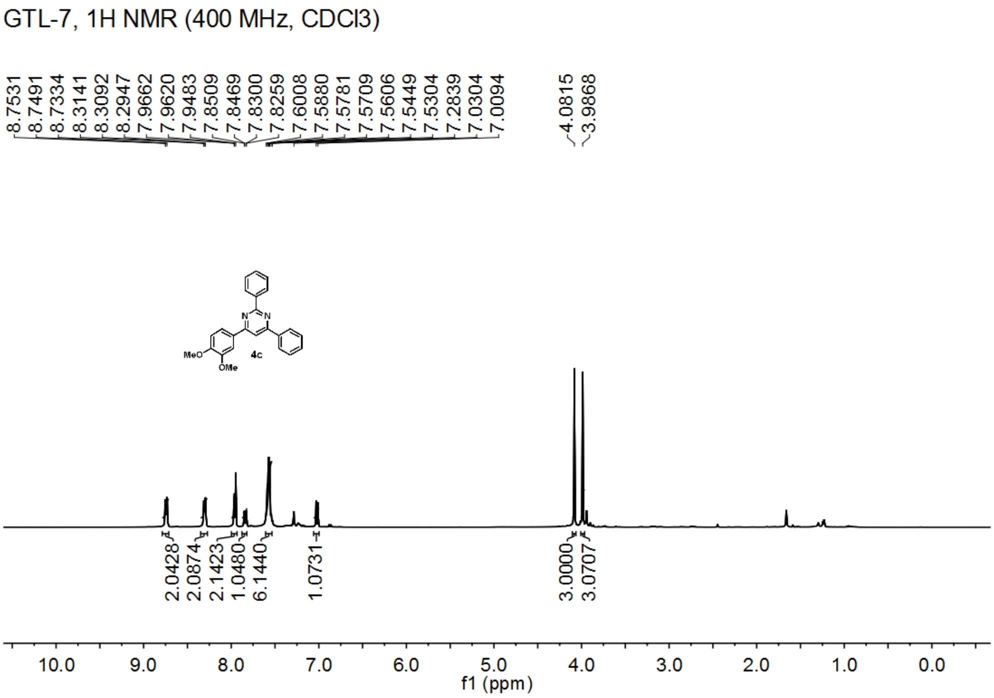

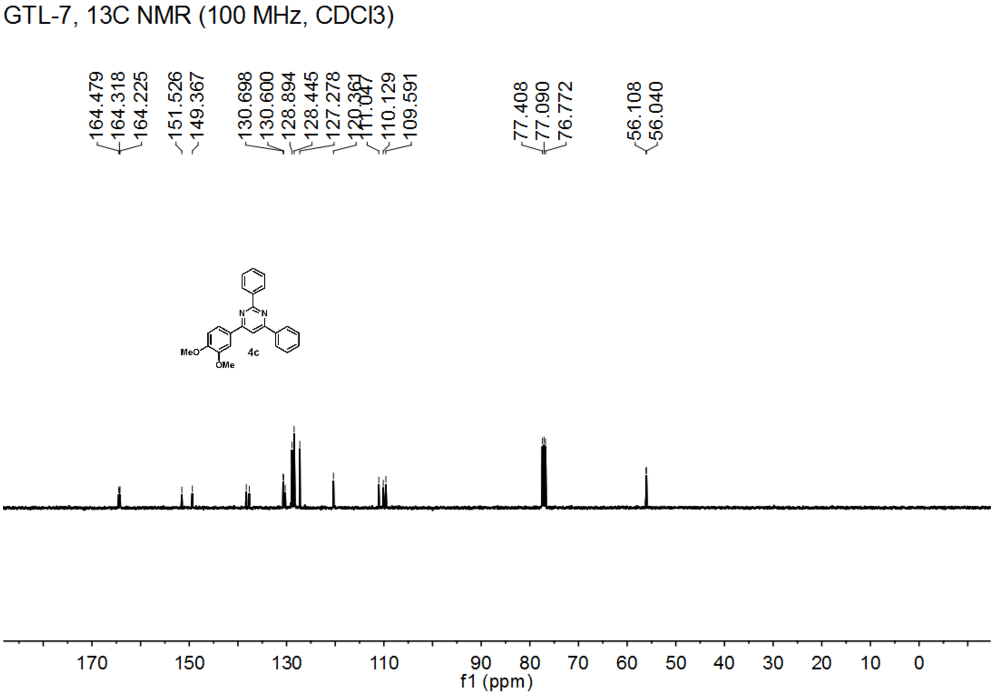

4-(3,4-dimethoxyphenyl)-2,6-diphenylpyrimidine (4c): 28 mg, yield 38%. White solid. ^{1}H NMR (400 MHz, $CDCl_3$) δ 8.74 (m, 2 H, aromatic CH), 8.31 (m, 2 H, aromatic CH), 7.97 (m, 2 H, aromatic CH), 7.84 (m, 1 H, aromatic CH), 7.57 (m, 6 H, aromatic CH), 7.02 (d, *J* = 8.4 Hz, 1 H, aromatic CH), 4.08 and 3.99 (s each, 3:3 H, 2×OCH_3). ^{13}C NMR (100 MHz, $CDCl_3$) δ 164.5, 164.3, 164.2, 151.5, 149.4, 138.3, 137.7 and 130.3 (Cq each), 130.7, 130.6, 128.9, 128.4, 127.3, 120.4, 111.1, 110.1 and 109.6 (CH), 56.1 and 56.0 (2×O*C*H_3).

Supplementary Fig. 6. ^{1}H NMR and ^{13}C NMR spectra for **4c** (Fig. 2 Entry 7).

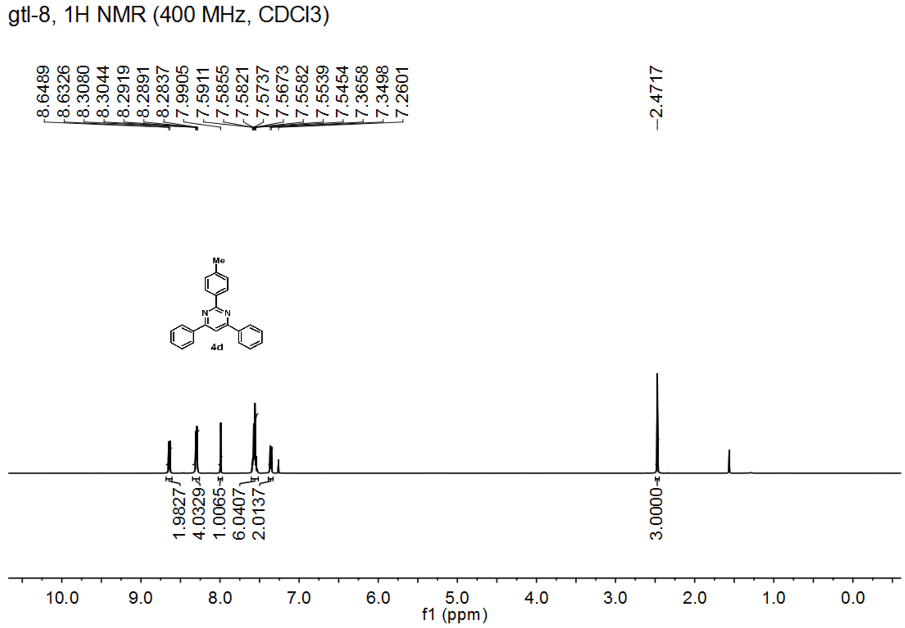

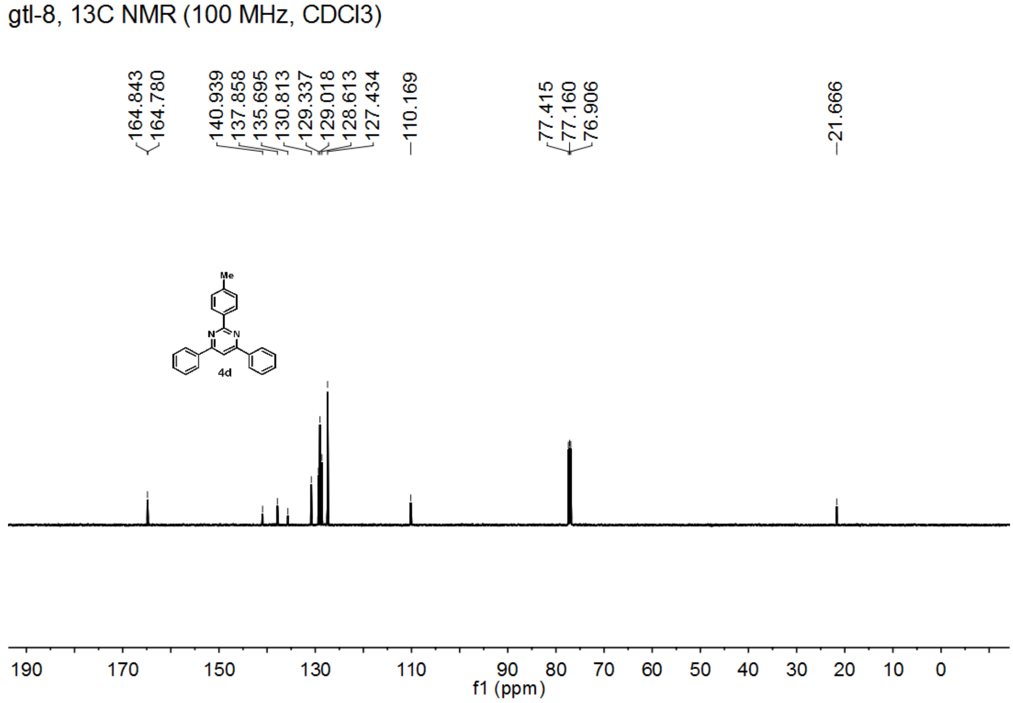

4,6-Diphenyl-2-(p-tolyl)pyrimidine (4d): 60 mg, yield 93%. White solid. ^{1}H NMR (400 MHz, $CDCl_3$) δ 8.64 (d, *J* = 8.2 Hz, 2 H, aromatic CH), 8.35-8.26 (m, 4 H, aromatic CH), 7.99 (s, 1 H, aromatic CH), 7.56 (m, 6 H, aromatic CH), 7.36 (d, *J* = 8.0 Hz, 2 H, aromatic CH), 2.47 (s, 3 H, CH_3). ^{13}C NMR (100 MHz, $CDCl_3$) δ 164.8, 164.8, 140.9, 137.9 and 135.7 (Cq each), 130.8, 129.3, 129.0, 128.6, 127.4 and 110.2 (CH), 21.7 (CH_3).

Supplementary Fig. 7. ^{1}H NMR and ^{13}C NMR spectra for **4d** (Fig.3 Entry 1).

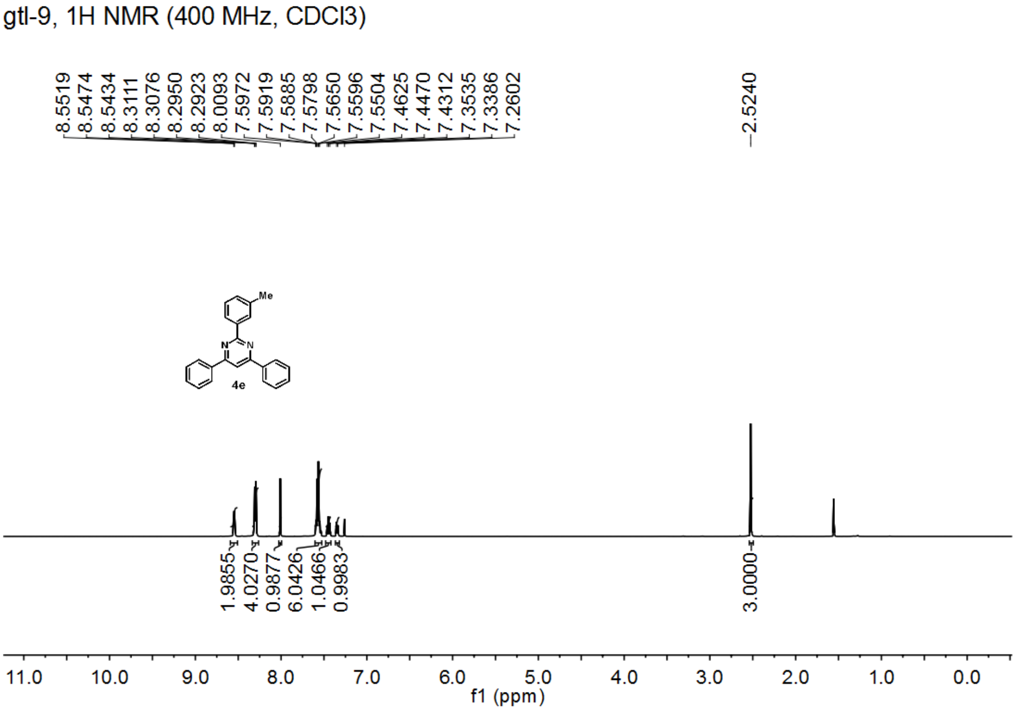

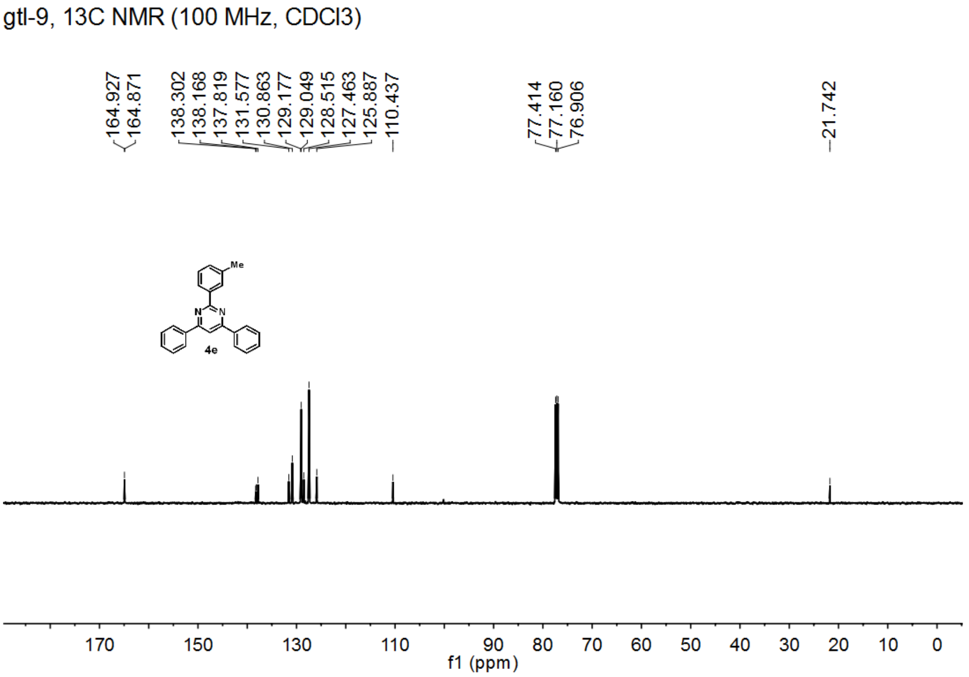

4,6-Diphenyl-2-(m-tolyl)pyrimidine (4e): 60 mg, yield 93%. White solid. ^{1}H NMR (400 MHz, $CDCl_3$) δ 8.55 (m, 2 H, aromatic CH), 8.30 (m, 4 H, aromatic CH), 8.01 (s, 1 H, aromatic CH), 7.58 (m, 6 H, aromatic CH), 7.45 (t, *J* = 7.8 Hz, 1 H, aromatic CH), 7.35 (d, *J* = 7.5 Hz, 1 H, aromatic CH), 2.52 (s, 3 H, CH_3). ^{13}C NMR (100 MHz, $CDCl_3$) δ 164.9, 138.3, 138.2, 137.8 and 131.6 (Cq each), 130.9, 129.2, 129.1, 128.5, 127.5, 125.9 and 110.4 (CH), 21.7 (CH_3).

Supplementary Fig. 8. ^{1}H NMR and ^{13}C NMR spectra for **4e** (Fig. 3 Entry 2).

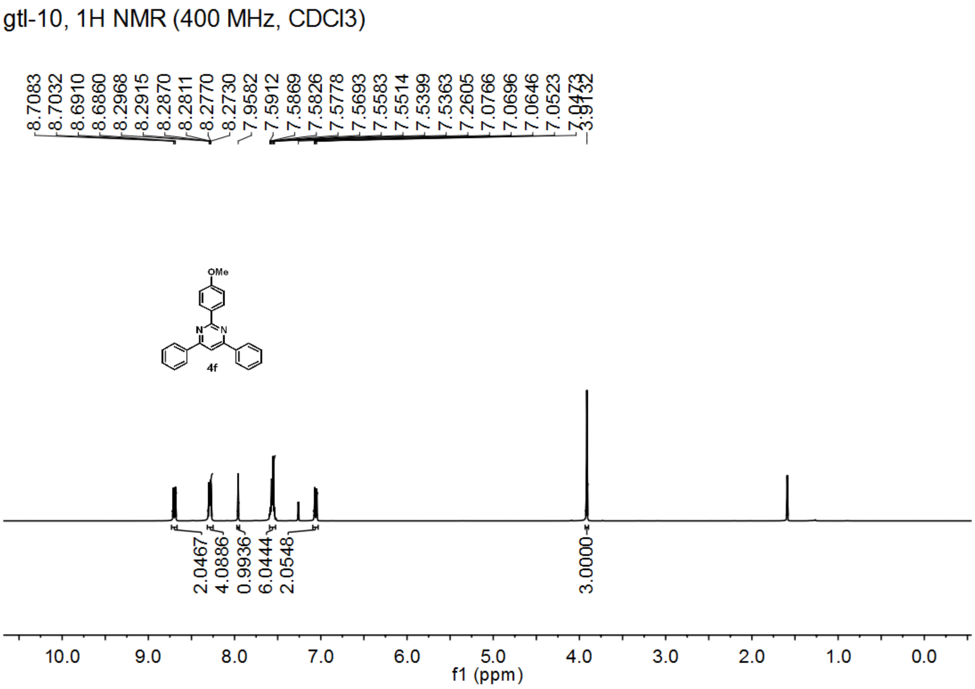

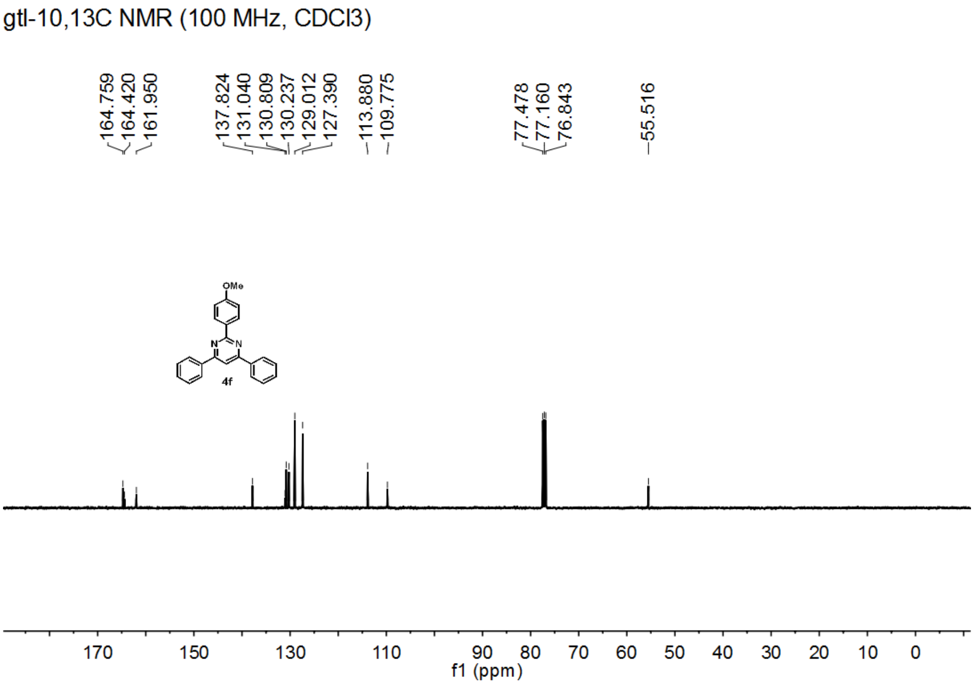

2-(4-Methoxyphenyl)-4,6-diphenylpyrimidine (4f): 62 mg, yield 92%. White solid. ^{1}H NMR (400 MHz, $CDCl_3$) δ 8.70 (m, 2 H, aromatic CH), 8.28 (m, 4 H, aromatic CH), 7.96 (s, 1 H, aromatic CH), 7.55 (m, 6 H, aromatic CH), 7.06 (m, 2 H, aromatic CH), 3.91 (s, 3 H, OCH_3). ^{13}C NMR (100 MHz, $CDCl_3$) δ 164.8, 164.4, 161.9, 137.8 and 131.0 (Cq each), 130.8, 130.2, 129.0, 127.4, 113.9 and 109.8 (CH), 55.5 (OCH_3).

Supplementary Fig. 9. ^{1}H NMR and ^{13}C NMR spectra for **4f** (Fig. 3 Entry 3).

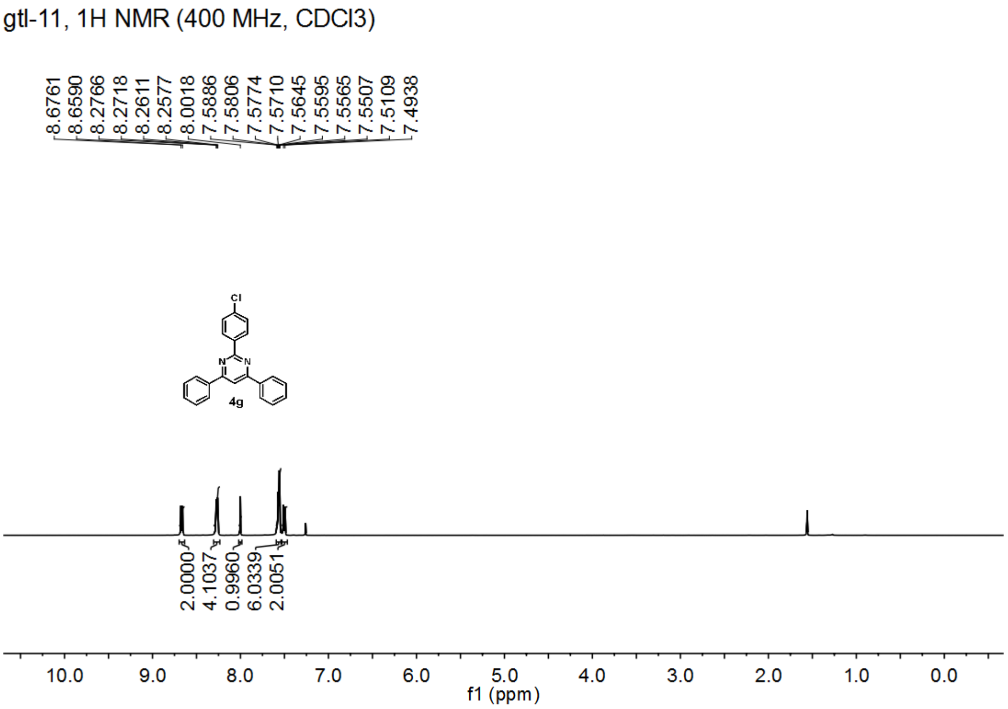

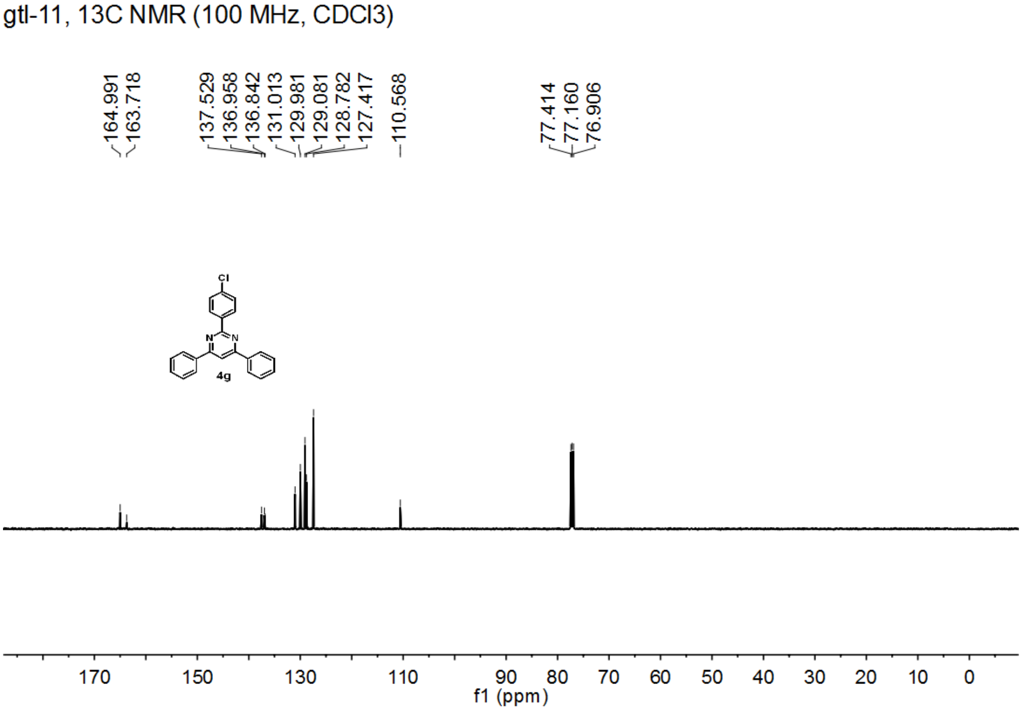

2-(4-Chlorophenyl)-4,6-diphenylpyrimidine (4g): 64 mg, yield 94%. White solid. ^{1}H NMR (400 MHz, $CDCl_3$) δ 8.67 (d, *J* = 8.5 Hz, 2 H, aromatic CH), 8.27 (m, 4 H, aromatic CH), 8.00 (s, 1 H, aromatic CH), 7.56 (m, 6 H, aromatic CH), 7.50 (d, *J* = 8.5 Hz, 2 H, aromatic CH). ^{13}C NMR (100 MHz, $CDCl_3$) δ 165.0, 163.7, 137.5, 137.0 and 136.8 (Cq each), 131.0, 130.0, 129.1, 128.8, 127.4 and 110.6 (CH).

Supplementary Fig.10. ^{1}H NMR and ^{13}C NMR spectra for **4g** (Fig. 3 Entry 4).

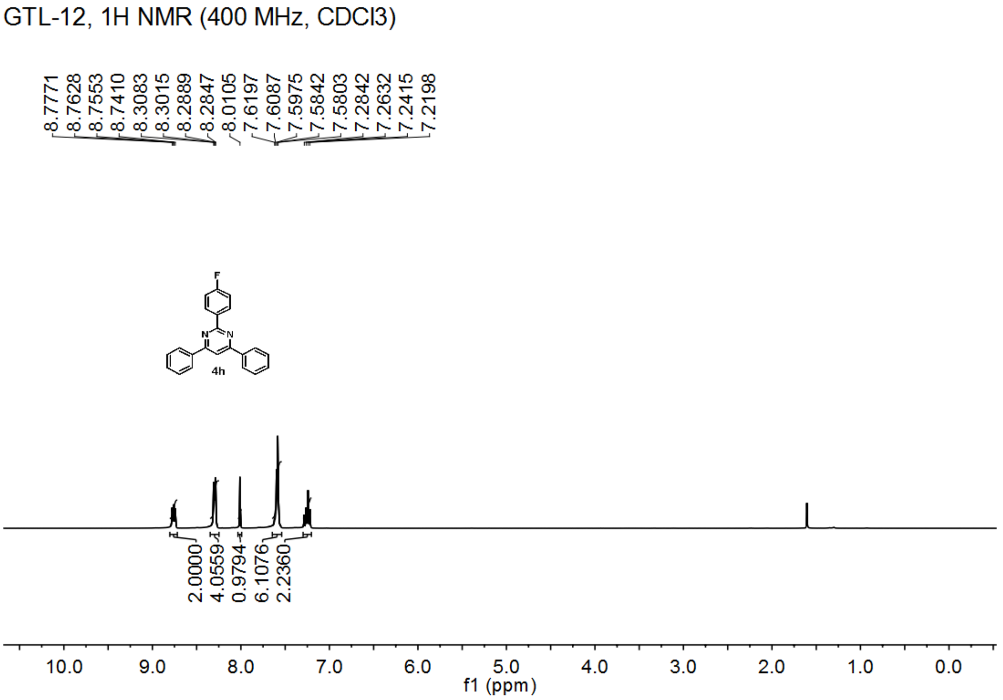

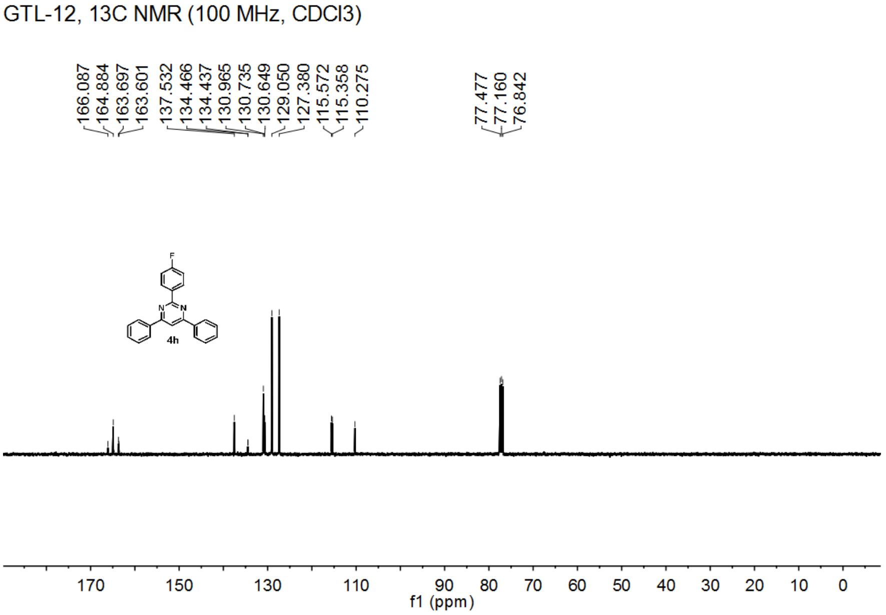

2-(4-Fluorophenyl)-4,6-diphenylpyrimidine (4h): 52 mg, yield 80%. White solid. ^{1}H NMR (400 MHz, $CDCl_3$) δ 8.76 (m, 2 H, aromatic CH), 8.30 (m, 4 H, aromatic CH), 8.01 (s, 1 H, aromatic CH), 7.65-7.54 (m, 6 H, aromatic CH), 7.25 (m, 2 H, aromatic CH). ^{13}C NMR (100 MHz, $CDCl_3$) δ 164.9, 164.8 (Cq, d, J = 248.6 Hz, *i*-C of C_6H_4F), 163.7, 137.5 and 134.4 (Cq, d, J = 2.9 Hz, *p*-C of C_6H_4F) (Cq each), 131.0, 130.7 (CH, d, J = 8.6 Hz, *m*-C of C_6H_4F), 129.1, 127.4, 115.4 (CH, d, J = 21.4 Hz, *o*-C of C_6H_4F) and 110.3 (CH).

Supplementary Fig. 11. ^{1}H NMR and ^{13}C NMR spectra for **4h** (Fig. 3, Entry 5).

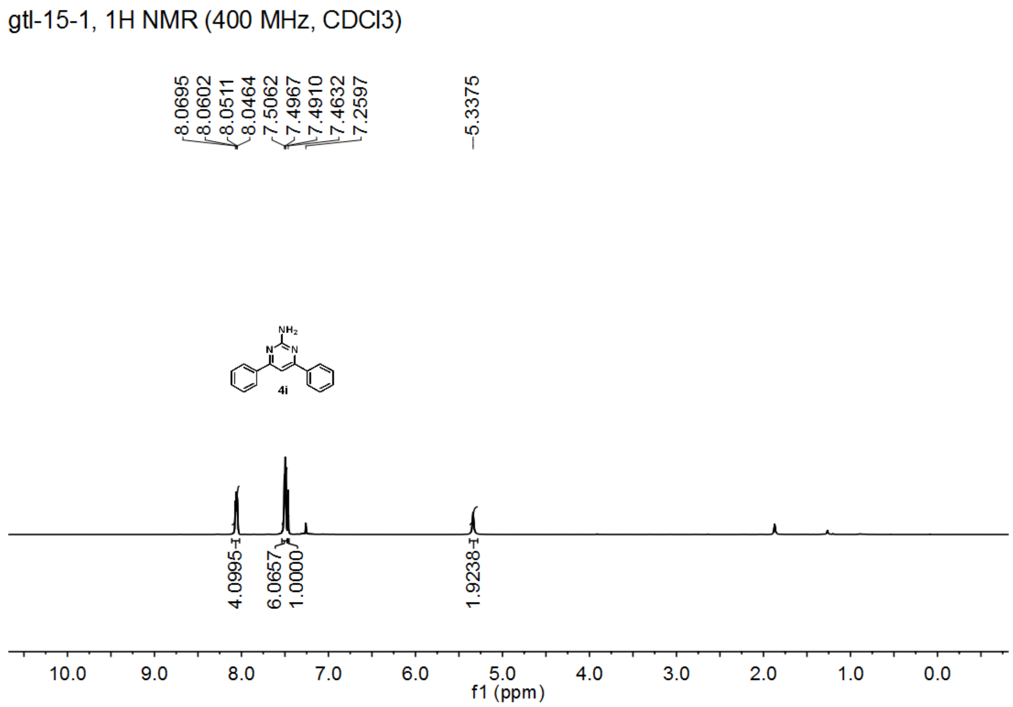

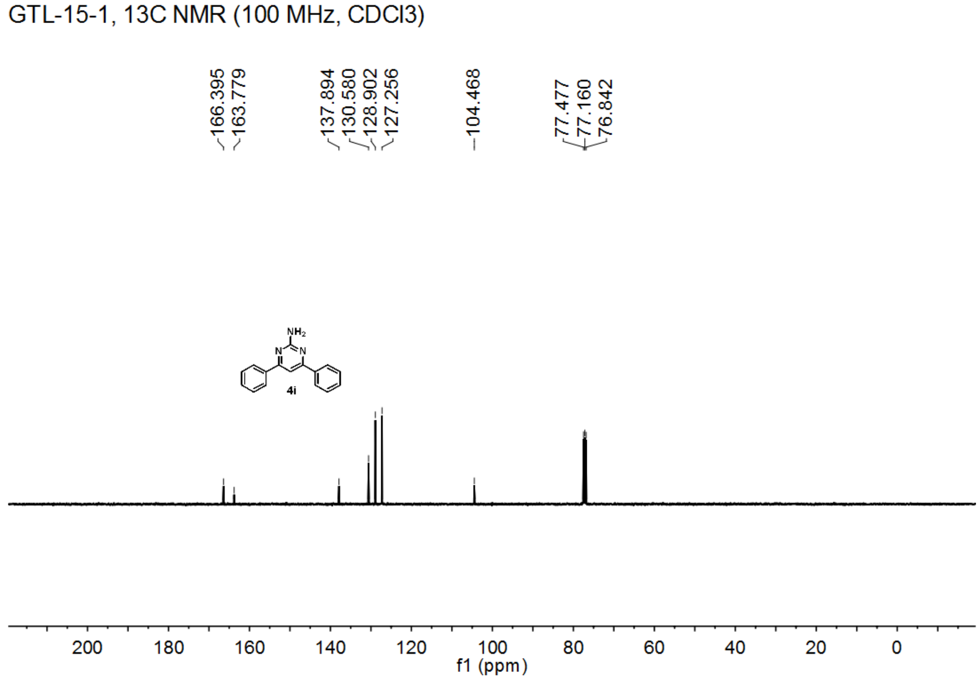

4,6-Diphenylpyrimidin-2-amine (4i): 41 mg, yield 83%. White solid. ^1H NMR (400 MHz, $CDCl_3$) δ 8.06 (m, 4 H, aromatic CH), 7.50 (m, 6 H, aromatic CH), 7.46 (s, 1 H, aromatic CH), 5.34 (s, 2 H, NH_2). ^{13}C NMR (100 MHz, $CDCl_3$) δ 166.4, 163.8 and 137.9 (Cq each), 130.6, 128.9, 127.3 and 104.4 (CH).

Supplementary Fig. 12. ^1H NMR and ^{13}C NMR spectra for **4i** (Fig. 3 Entry 6).

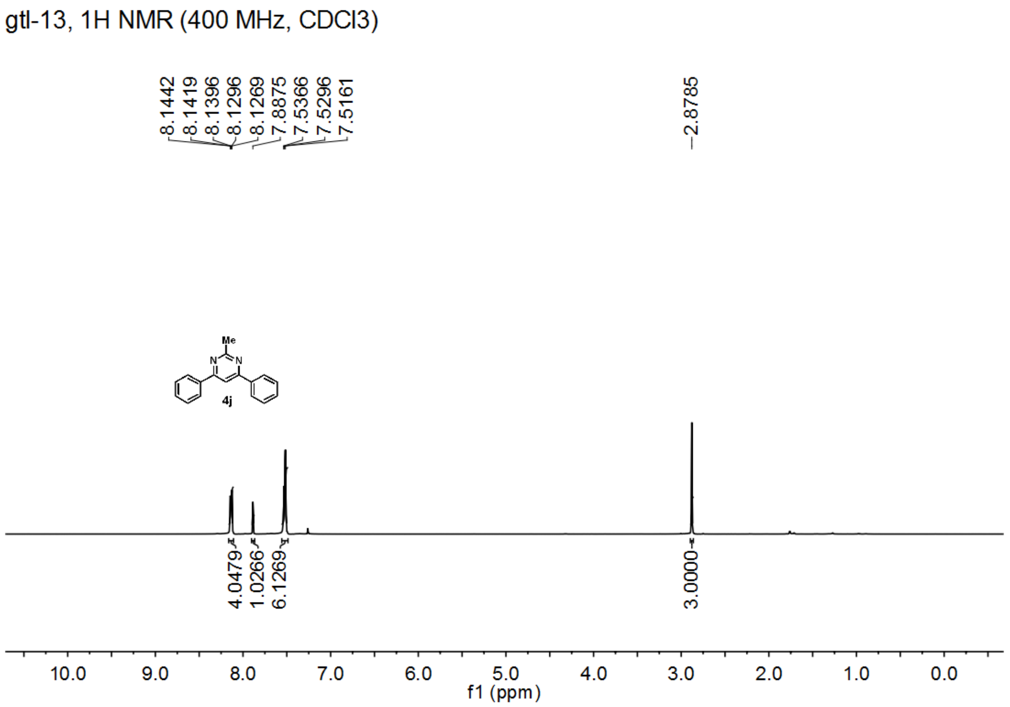

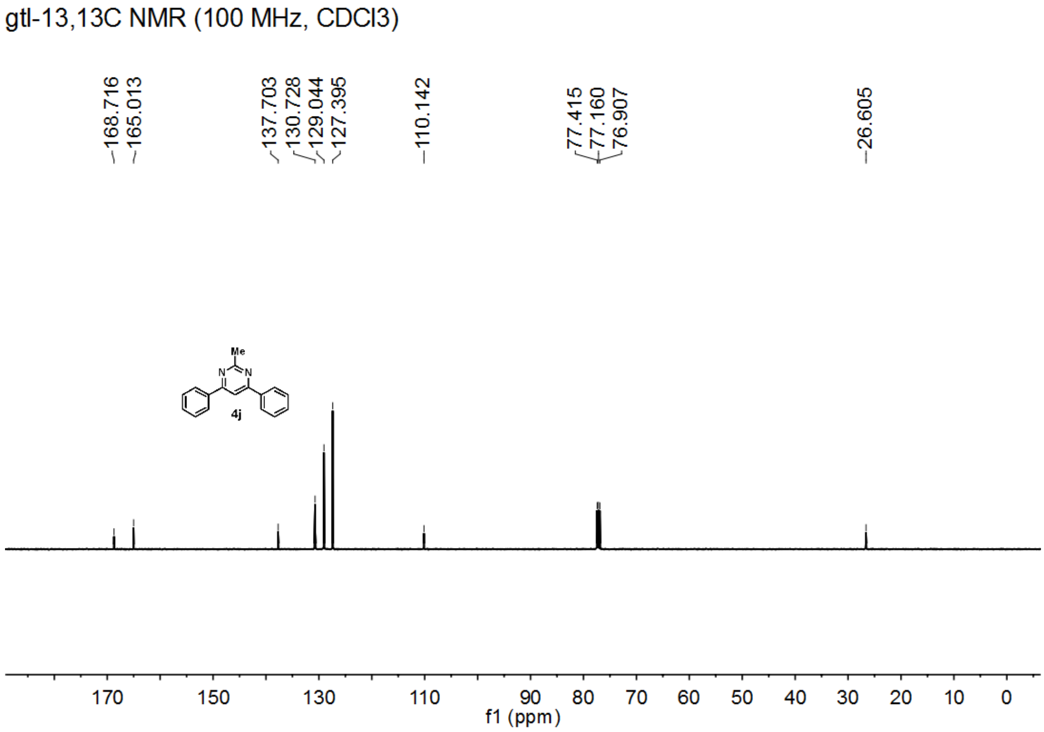

2-Methyl-4,6-diphenylpyrimidine (4j): 32 mg, yield 64%. White solid. ^{1}H NMR (400 MHz, $CDCl_3$) δ 8.14 (m, 4 H, aromatic CH), 7.89 (s, 1 H, aromatic CH), 7.53 (m, 6 H, aromatic CH), 2.88 (s, 3 H, CH_3). ^{13}C NMR (100 MHz, $CDCl_3$) δ 168.7, 165.0 and 137.7 (Cq each), 130.7, 129.0, 127.4 and 110.1 (CH), 26.6 (CH_3).

Supplementary Fig. 13. ^{1}H NMR and ^{13}C NMR spectra for **4j** (Fig. 3 Entry 7).

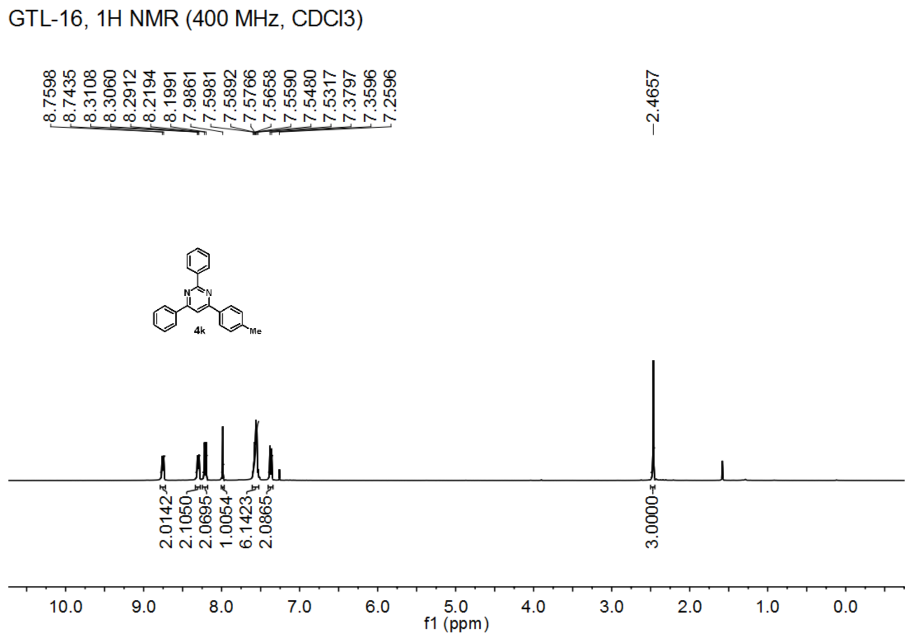

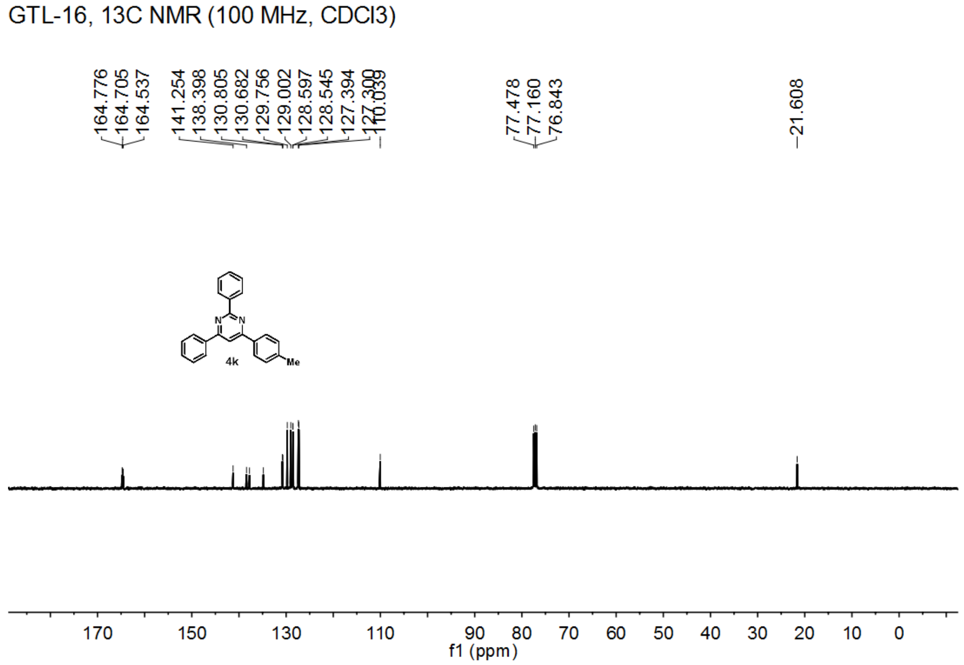

2,4-Diphenyl-6-(p-tolyl)pyrimidine (4k): 50 mg, yield 78%. White solid. ^{1}H NMR (400 MHz, $CDCl_3$) δ 8.75 (d, J = 6.5 Hz, 2 H, aromatic CH), 8.30 (m, 2 H, aromatic CH), 8.21 (d, J = 8.1 Hz, 2 H, aromatic CH), 7.99 (s, 1 H, aromatic CH), 7.61-7.52 (m, 6 H, aromatic CH), 7.37 (d, J = 8.1 Hz, 2 H, aromatic CH), 2.47 (s, 3 H, CH_3). ^{13}C NMR (100 MHz, $CDCl_3$) δ 164.8, 164.7, 164.5, 141.2, 138.4, 137.8 and 134.8 (Cq each), 130.8, 130.7, 129.8, 129.0, 128.6, 128.6, 127.4, 127.3 and 110.0 (CH), 21.6 (CH_3).

Supplementary Fig. 14. ^{1}H NMR and ^{13}C NMR spectra for **4k** (Fig.3. Entry 8).

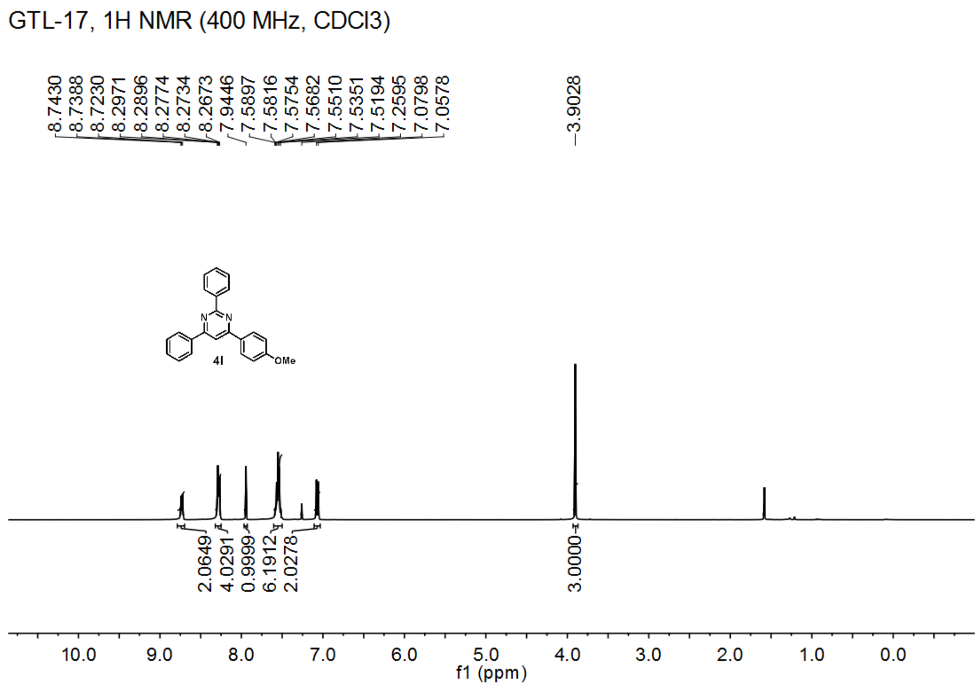

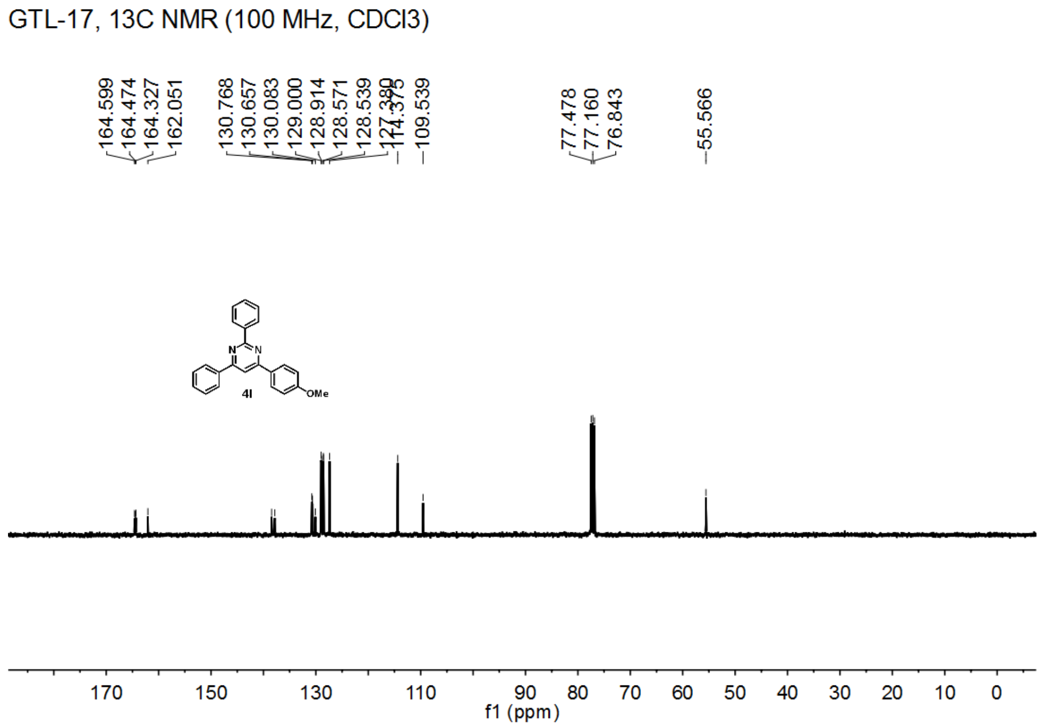

4-(4-Methoxyphenyl)-2,6-diphenylpyrimidine (4l): 52 mg, yield 78%. White solid. ^{1}H NMR (400 MHz, $CDCl_3$) δ 8.79-8.70 (m, 2 H, aromatic CH), 8.28 (m, 4 H, aromatic CH), 7.94 (s, 1 H, aromatic CH), 7.60-7.50 (m, 6 H, aromatic CH), 7.07 (d, J = 8.8 Hz, 2 H, aromatic CH), 3.90 (s, 3 H, OMe). ^{13}C NMR (100 MHz, $CDCl_3$) δ 164.6, 164.5, 164.3, 162.1, 138.4, 137.8 and 130.1 (Cq each), 130.8, 130.7, 129.0, 128.9, 128.6, 128.5, 127.4, 114.4 and 109.5 (CH), 55.6 (OCH_3).

Supplementary Fig.15. ^{1}H NMR and ^{13}C NMR spectra for **4l** (Fig. 3 Entry 9).

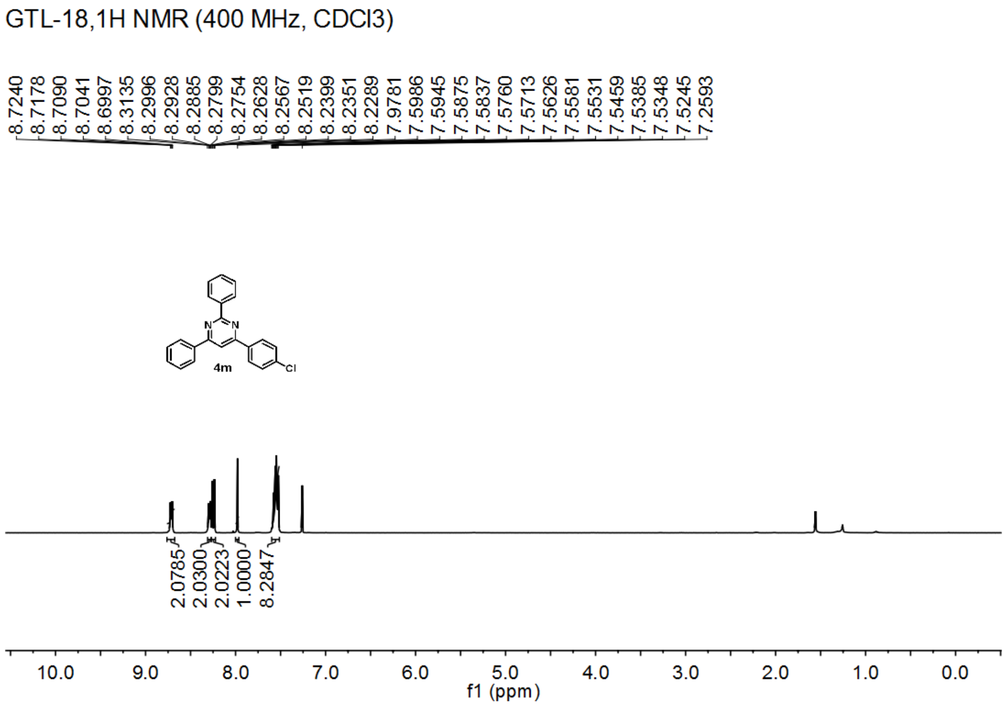

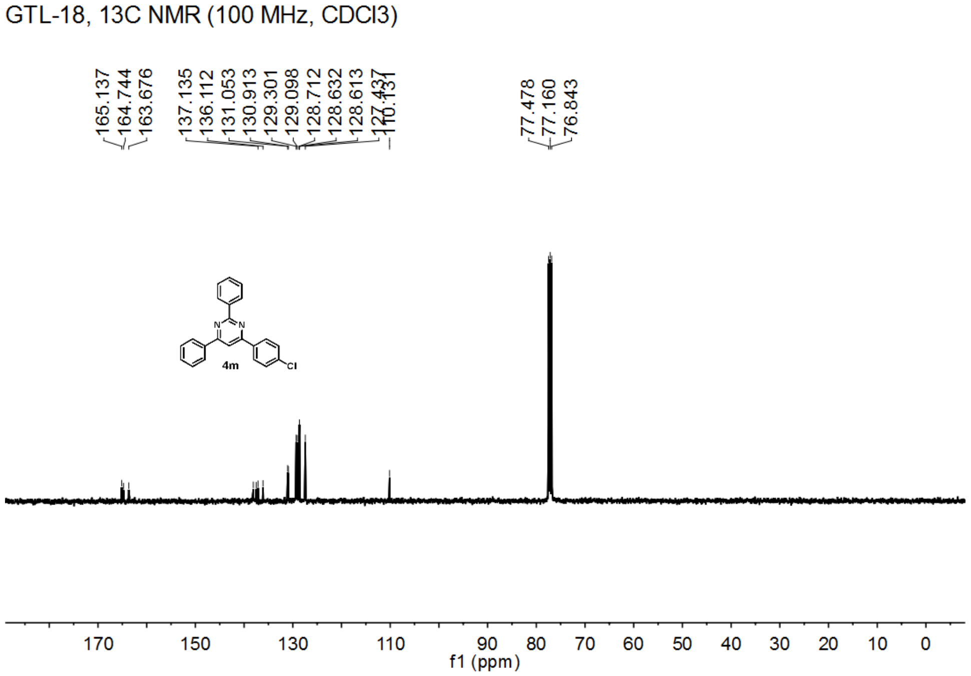

4-(4-Chlorophenyl)-2,6-diphenylpyrimidine (4m): 63 mg, yield 92%. White solid. ^{1}H NMR (400 MHz, $CDCl_3$) δ 8.71 (m, 2 H, aromatic CH), 8.28 (m, 2 H, aromatic CH), 8.24 (d, *J* = 8.6 Hz, 2 H, aromatic CH), 7.97 (s, 1 H, aromatic CH), 7.56 (m, 8 H, aromatic CH). ^{13}C NMR (100 MHz, $CDCl_3$) δ 165.1, 164.7, 163.7, 138.1, 137.5, 137.1 and 136.1 (Cq each), 131.1, 130.9, 129.3, 129.1, 128.7, 128.6, 128.6, 127.4 and 110.1 (CH).

Supplementary Fig. 16. ^{1}H NMR and ^{13}C NMR spectra for **4m** (Fig. 3 Entry 10).

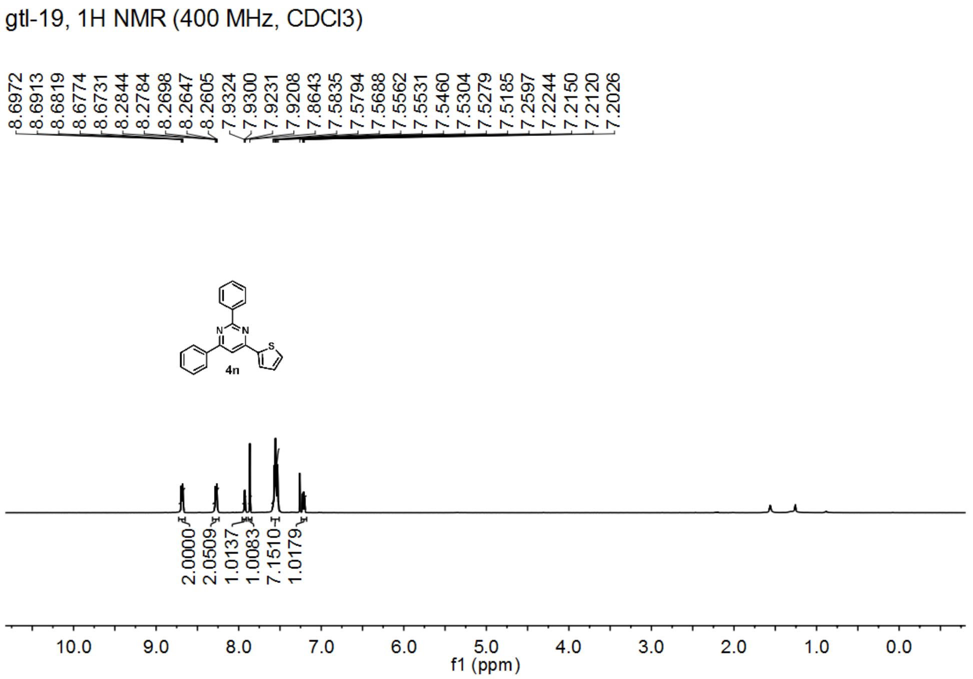

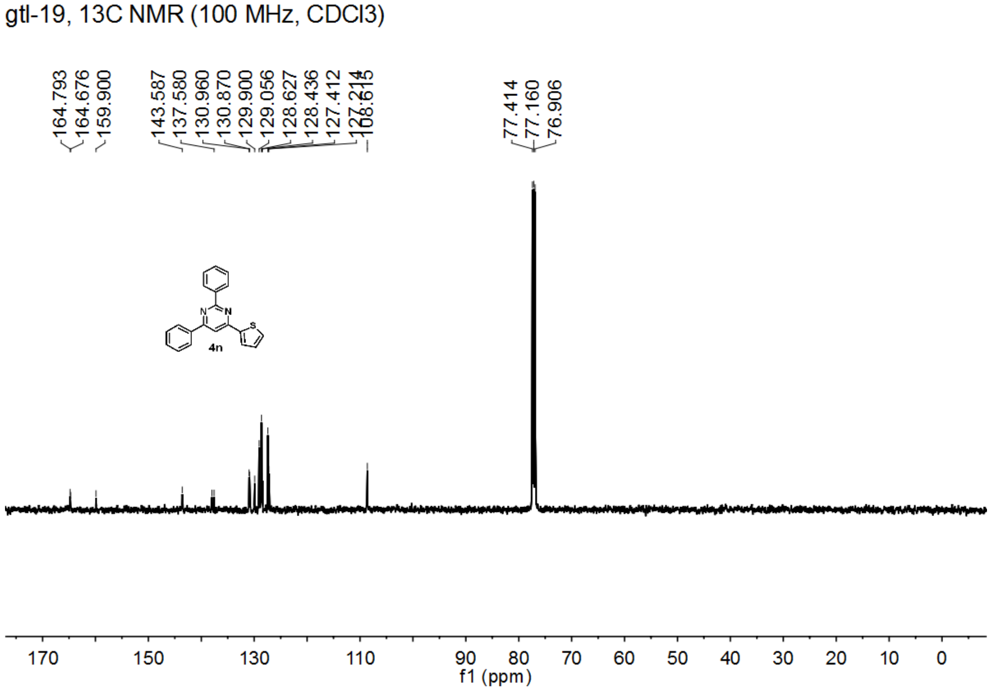

2,4-Diphenyl-6-(thiophen-2-yl)pyrimidine (4n): 50 mg, yield 80%. White solid. ^{1}H NMR (400 MHz, $CDCl_3$) δ 8.68 (dd, *J* = 7.8, 1.7 Hz, 2 H, aromatic CH), 8.27 (dd, *J* = 7.7, 1.7 Hz, 2 H, aromatic CH), 7.93 (m, 1 H, thienyl CH), 7.86 (s, 1 H, aromatic CH), 7.59-7.50 (m, 7 H, aromatic CH and thienyl CH), 7.21 (m, 1 H, thienyl CH). ^{13}C NMR (100 MHz, $CDCl_3$) δ 164.8, 164.7, 159.9, 143.6, 138.0 and 137.6 (Cq each), 131.0, 130.9, 129.9, 129.1, 128.6, 128.4, 127.4, 127.2 and 108.6 (CH).

Supplementary Fig. 17. ^{1}H NMR and ^{13}C NMR spectra for **4n** (Fig. 3 Entry 11).

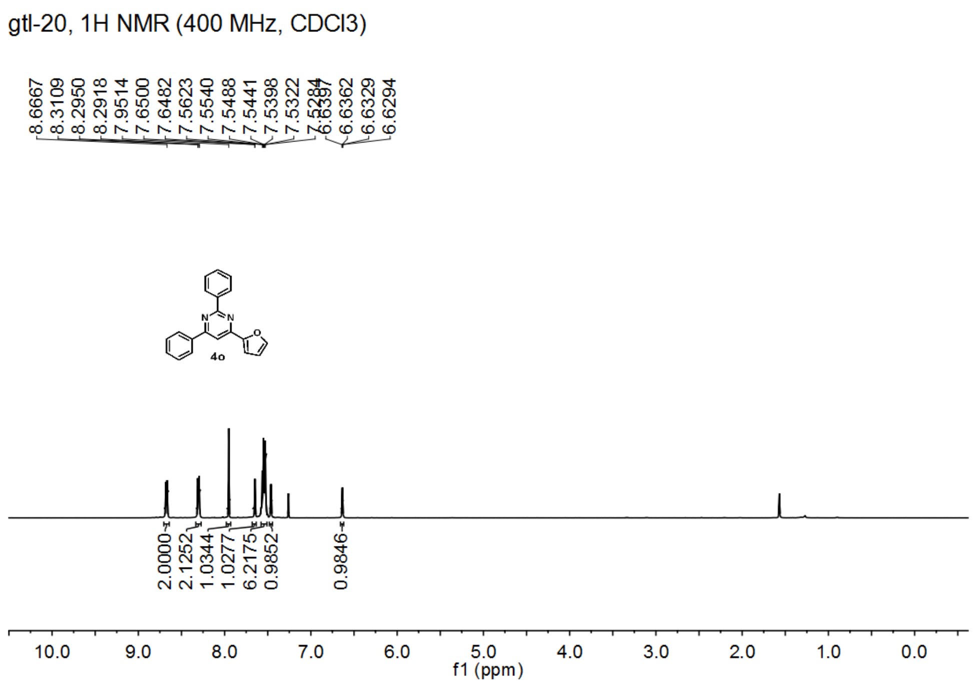

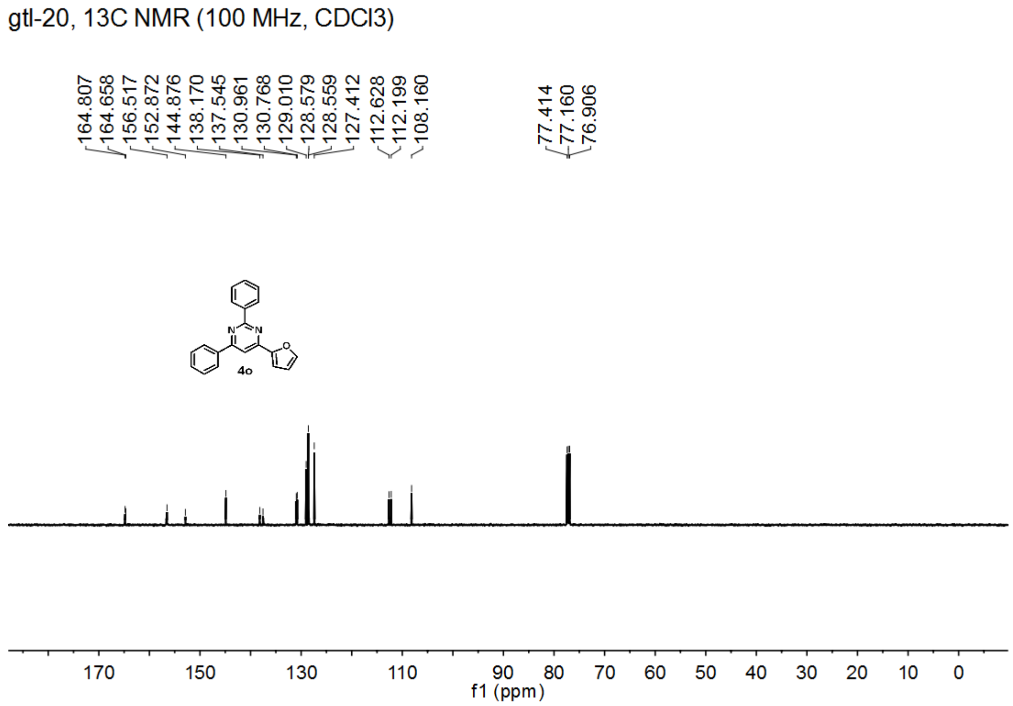

4-(Furan-2-yl)-2,6-diphenylpyrimidine (4o): 42 mg, yield 71%. White solid. ^{1}H NMR (400 MHz, $CDCl_3$) δ 8.67 (dd, *J* = 7.7, 1.8 Hz, 2 H, aromatic CH), 8.30 (m, 2 H, aromatic CH), 7.95 (s, 1 H, aromatic CH), 7.65 (d, *J* = 0.9 Hz, 1 H, furyl CH), 7.55 (m, 6 H, aromatic CH), 7.46 (d, *J* = 3.4 Hz, 1 H, furyl CH), 6.63 (dd, *J* = 3.4, 1.7 Hz, 1 H, furyl CH). ^{13}C NMR (100 MHz, $CDCl_3$) δ 164.8, 164.7, 156.5, 152.9, 138.2 and 137.5 (Cq each), 144.9, 131.0, 130.8, 129.0, 128.6, 128.6, 127.4, 112.6, 112.2 and 108.2 (CH).

Supplementary Fig. 18. ^{1}H NMR and ^{13}C NMR spectra for **4o** (Fig. 3 Entry 12).

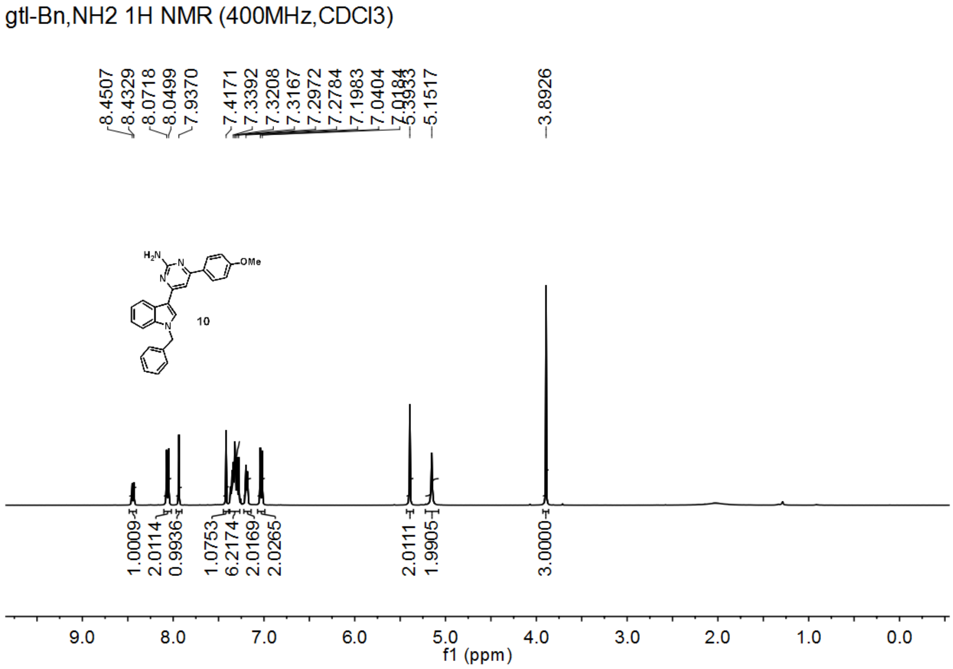

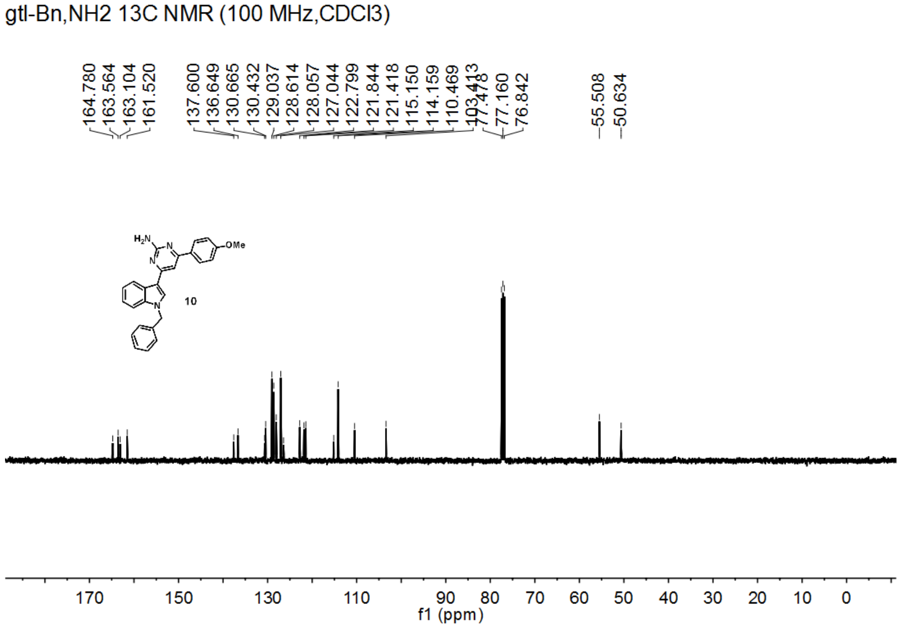

4-(1-Benzyl-1H-indol-3-yl)-6-(4-methoxyphenyl)pyrimidin-2-amine (10): 56 mg, yield 69%. White solid. ^{1}H NMR (400 MHz, $CDCl_3$) δ 8.44 (d, J = 7.1 Hz, 1 H, aromatic CH), 8.06 (d, J = 8.8 Hz, 2 H, aromatic CH), 7.94 (s, 1 H, aromatic CH), 7.42 (s, 1 H, aromatic CH), 7.38-7.27 (m, 6 H, aromatic CH), 7.19 (d, J = 6.5 Hz, 2 H, aromatic CH), 7.03 (d, J = 8.8 Hz, 2 H, aromatic CH), 5.39 (s, 2 H, $PhCH_2$), 5.15 (s, 2 H, NH_2), 3.89 (s, 3 H, OCH_3). ^{13}C NMR (100 MHz, $CDCl_3$) δ 164.8, 163.6, 163.1, 161.5, 137.6, 136.6, 130.7, 126.4 and 115.2 (Cq each), 130.4, 129.0, 128.6, 128.1, 127.0, 122.8, 121.8, 121.4, 114.2, 110.5 and 103.4 (CH), 55.5 ($PhCH_2$), 50.6 (OCH_3).

Supplementary Fig. 19. ^{1}H NMR and ^{13}C NMR spectra for **10** (Fig. 8).

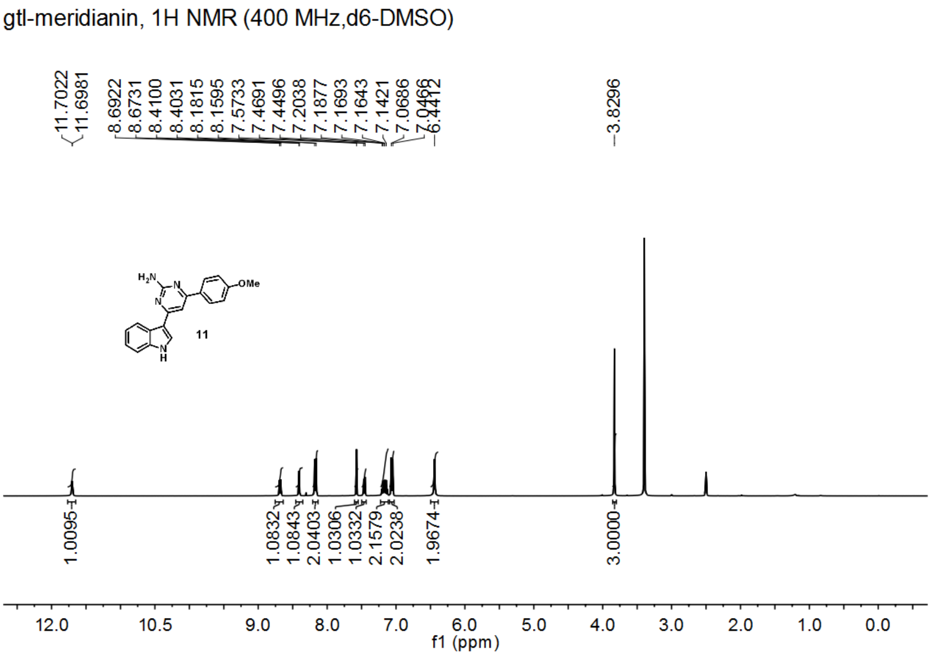

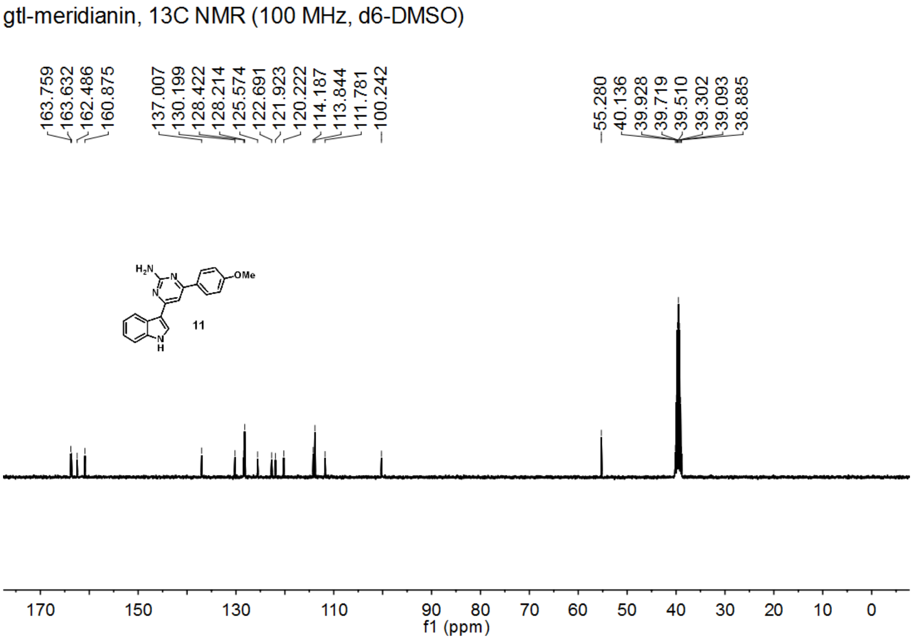

4-(1H-indol-3-yl)-6-(4-methoxyphenyl)pyrimidin-2-amine (11): 22 mg, yield 70%. White solid. ^{1}H NMR (400 MHz, d_6-DMSO) δ 11.70 (d, J = 1.6 Hz, 1 H, NH), 8.68 (d, J = 7.6 Hz, 1 H, aromatic CH), 8.41 (d, J = 2.8 Hz, 1 H, aromatic CH), 8.17 (d, J = 8.8 Hz, 2 H, aromatic CH), 7.57 (s, 1 H, aromatic CH), 7.46 (d, J = 7.8 Hz, 1 H, aromatic CH), 7.17 (m, 2 H, aromatic CH), 7.06 (d, J = 8.8 Hz, 2 H, aromatic CH), 6.44 (s, 2 H, indolyl CH), 3.83 (s, 3 H, OCH_3). ^{13}C NMR (100 MHz, DMSO) δ 163.8, 163.6, 162.5, 160.9, 137.0, 130.2, 125.6 and 114.2 (Cq each), 128.4, 128.2, 122.7, 121.9, 120.2, 113.8, 111.8 and 100.2 (CH), 55.3 (OCH_3).

Supplementary Fig. 20. ^{1}H NMR and ^{13}C NMR spectra for **11** (Fig. 8).

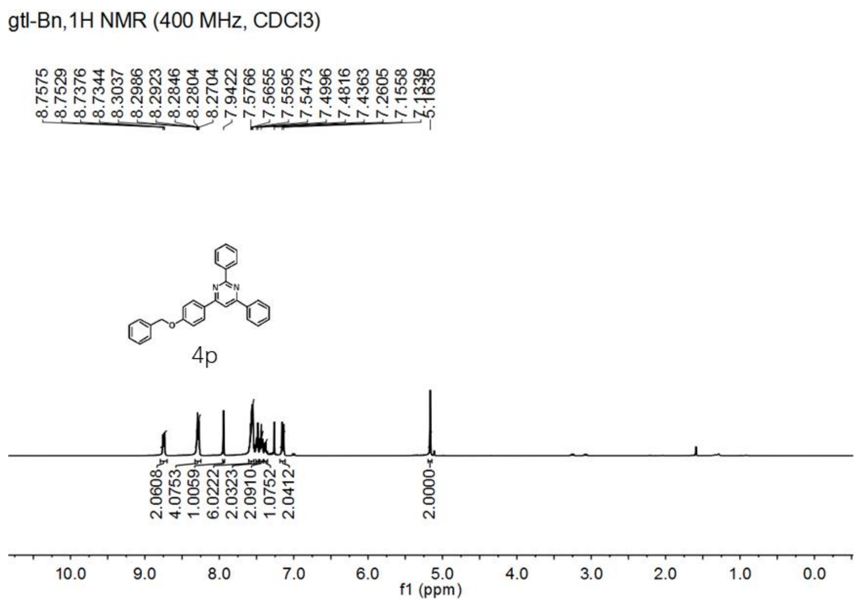

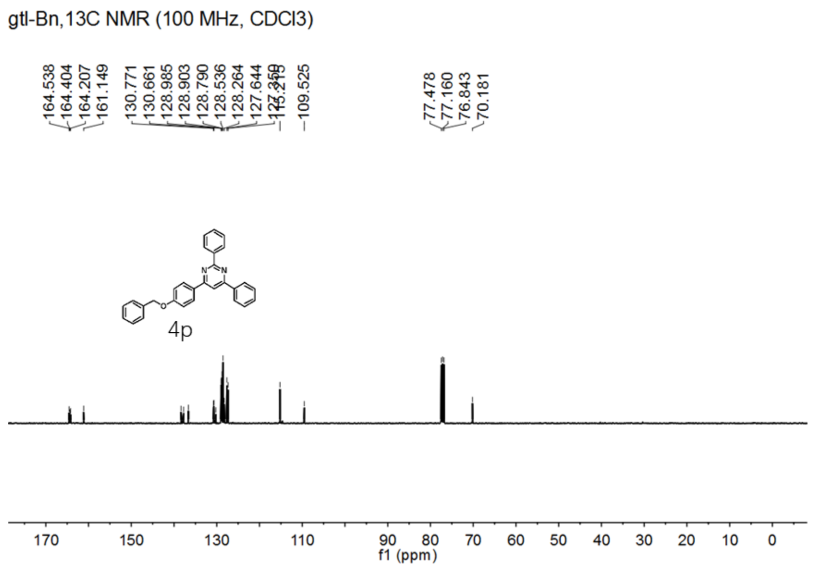

4-(4-(benzyloxy)phenyl)-2,6-diphenylpyrimidine (4p): 99 mg, yield 92%. White solid, m.p.: 104-106 ^{o}C. ^{1}H NMR (400 MHz, CDCl3) δ 8.75 (m, 2 H, aromatic CH), 8.33-8.25 (m, 4 H, aromatic CH), 7.94 (s, 1 H, aromatic CH), 7.61-7.53 (m, 6 H, aromatic CH), 7.49 (d, *J* = 7.2 Hz, 2 H, aromatic CH), 7.44 (t, *J* = 7.3 Hz, 2 H, aromatic CH), 7.38 (d, *J* = 7.1 Hz, 1 H, aromatic CH), 7.14 (d, *J* = 8.8 Hz, 2 H, aromatic CH), 5.16 (s, 2 H, PhC*H₂*). ^{13}C{^{1}H} (100 MHz, $CDCl_3$) δ 164.5, 164.4, 164.2, 161.1, 138.4, 137.8, 136.6 and 130.2 (Cq each), 130.8, 130.7, 129.0, 128.9, 128.8, 128.5, 128.3, 127.6, 127.3, 115.2 and 109.5 (CH), 70.2 (Ph*C*H$_2$). HRMS Calcd for $C_{29}H_{23}N_2O$[M+H]$^+$: 415.1810; Found: 415.1814.

Supplementary Fig. 21. ^{1}H NMR and ^{13}C NMR spectra for **4p** (Supplementary Fig. 1.).

1.5. Details of DFT calculations

All calculations were performed at ωB97X-D/6-311+G(d,p) level using Gaussian 09 program.[5,6] In order to verify the reliability of the DFT calculations used in this work, the M06-2X/6-311+G(d,p)[7] was used to investigate the C-O bond cleavage of the β-O-4 model compound, but no qualitatively different results were obtained (Supplementary Data 3). The solvation effect of tert-amyl alcohol (ε=5.78) was simulated by the SMD continuum solvent mode.[8] All transition states were further confirmed by vibrational analysis and characterized by the only one imaginary frequency. Intrinsic reaction coordinates (IRC) calculations were performed in order to confirm intermediates along the reaction pathways.[9] All energies discussed in the following parts are Gibbs free energies calculated at 298.15 K unless otherwise stated.

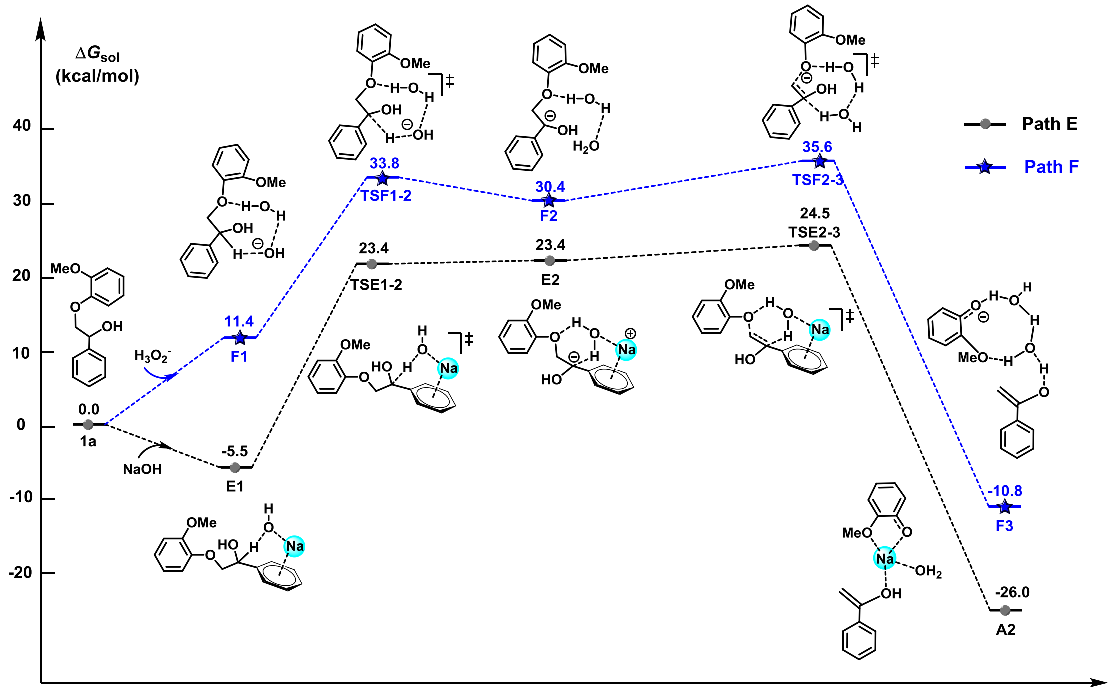

Supplementary Fig. 22. Other possible pathways (path E and path F) for the cleavage of the C-O bond of β-O-4 model compound **1a** (unit: kcal/mol).

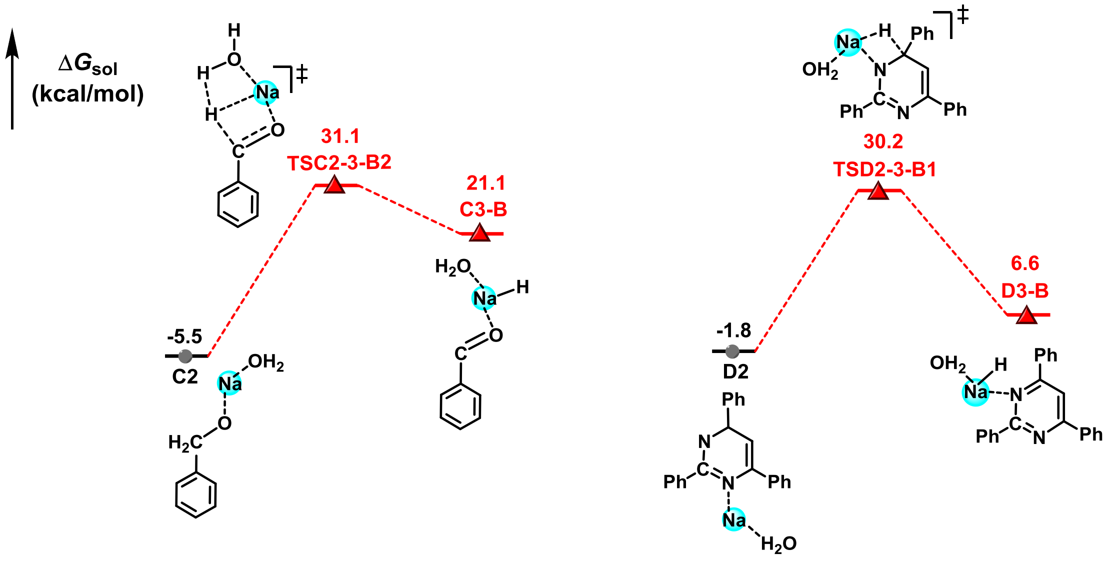

Supplementary Fig. 23. Other possible pathways for hydrogen-acceptorless dehydrogenation (unit: kcal/mol).

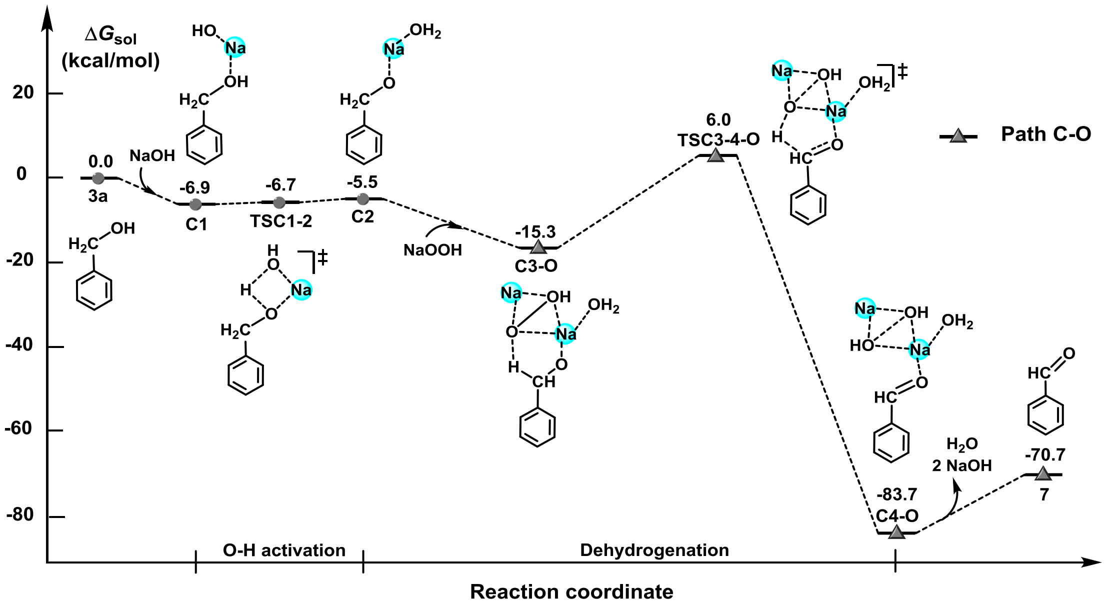

Supplementary Fig. 24. The Gibbs free energy profiles for the dehydrogenation of benzyl alcohol **3a** using by-product NaOOH as hydrogen acceptors (unit: kcal/mol).

2. Supplementary References

1 Nichols, J. M., Bishop, L. M., Bergman, R. G. & Ellman, J. A. Catalytic C−O bond cleavage of 2-aryloxy-1-arylethanols and its application to the depolymerization of lignin-related polymers. *J. Am. Chem. Soc.* **132**, 12554-12555 (2010).

2 Galkin, M. V., Dahlstrand, C. & Samec, J. S. M. Mild and robust redox-neutral Pd/C-catalyzed lignol β-O-4 bond cleavage through a low-energy-barrier pathway. *ChemSusChem* **8**, 2187-2192 (2015).

3 Liu, Y. Li, C. Z., Miao, W., Tang, W., Xue, D., Li, C., Zhang, B., Xiao, J., Wang, A., Zhang, T. & Wang, C. Mild redox-neutral depolymerization of lignin with a binuclear Rh complex in water. *ACS Catal.* **9**, 4441-4447 (2019).

4 Hanson, S. K., Wu, R. & Silks, L. A. P. C-C or C-O bond cleavage in a phenolic lignin model compound: selectivity depends on vanadium catalyst. *Angew. Chem. Int. Ed.* **51**, 3410-3413 (2012).

5 Gaussian 09 v. Gaussian 09, Revision B. 01 (Gaussian, Inc,Wallingford., CT 2010).

6 Chai, J.-D. & Head-Gordon, M. Long-range corrected hybrid density functionals with damped atom-atom dispersion corrections. *Phys. Chem. Chem. Phys.* **10**, 6615-6620 (2008).

7 Zhao, Y. & Truhlar, D. G. The M06 suite of density functionals for main group thermochemistry, thermochemical kinetics, noncovalent interactions, excited states, and transition elements: two new functionals and systematic testing of four M06-class functionals and 12 other functionals. *Theor. Chem. Acc.* **120**, 215-241 (2007).

8 Marenich, A. V., Cramer, C. J. & Truhlar, D. G. Universal solvation model based on solute electron density and on a continum model of the solvent defined by the bulk dielectric constant and atomic surface tensions. *J. Phys. Chem. B.* **113**, 6378-6396 (2009).

9 Hratchian, H. P.& Schlegel, H. B. Accurate reaction paths using a hessian based predictor-corrector Integrator. *J. Chem. Phys.* **120**, 9918-9924 (2004).
