# Supplementary material for: Transition-metal-free synthesis of pyrimidines from lignin β-O-4 segments via a one-pot multi-component reaction
Source: Nat Commun. 2022 Jun 11;13:3365. doi: 10.1038/s41467-022-30815-5 (PMC9188570; doi:10.1038/s41467-022-30815-5)
Supplement: Supplementary file 3 — Description of Additional Supplementary Files [file 41467_2022_30815_MOESM3_ESM.docx]

**Supplementary Data Legends/Captions**

**Supplementary Data 1.**

The calculated absolute electronic energies (*E*, in a.u.), thermal free energies (*G*, in a.u.), and relative Gibbs energies (Δ*G*, in kcal/mol) (Calculated at 298.15 K and 1 atm).

**Supplementary Data 2.**

Calculated imaginary frequencies of transition states at ωB97X-D/6-311+G(d,p) level.

**Supplementary Data 3.**

Atomic cartesian coordinates of intermediates and transition states (presented in Å).
